# The MqsRA Toxin-Antitoxin System from *Xylella fastidiosa* Plays a Key Role in Bacterial Fitness, Pathogenicity, and Persister Cell Formation

**DOI:** 10.3389/fmicb.2016.00904

**Published:** 2016-06-10

**Authors:** Marcus V. Merfa, Bárbara Niza, Marco A. Takita, Alessandra A. De Souza

**Affiliations:** ^1^Instituto Agronômico, Centro de Citricultura Sylvio MoreiraCordeirópolis, Brazil; ^2^Departamento de Genética, Evolução e Bioagentes, Universidade Estadual de CampinasCampinas, Brazil

**Keywords:** plant pathogen, biofilm, toxin-antitoxin system, persister cell, copper stress

## Abstract

Through the formation of persister cells, bacteria exhibit tolerance to multidrug and other environmental stresses without undergoing genetic changes. The toxin-antitoxin (TA) systems are involved in the formation of persister cells because they are able to induce cell dormancy. Among the TA systems, the MqsRA system has been observed to be highly induced in persister cells of *Xylella fastidiosa* (causal agent of citrus variegated chlorosis—CVC) activated by copper stress, and has been described in *Escherichia coli* as related to the formation of persister cells and biofilms. Thus, we evaluated the role of this TA system in *X. fastidiosa* by overexpressing the MqsR toxin, and verified that the toxin positively regulated biofilm formation and negatively cell movement, resulting in reduced pathogenicity in citrus plants. The overexpression of MqsR also increased the formation of persister cells under copper stress. Analysis of the gene and protein expression showed that this system likely has an autoregulation mechanism to express the toxin and antitoxin in the most beneficial ratio for the cell to oppose stress. Our results suggest that this TA system plays a key role in the adaptation and survival of *X. fastidiosa* and reveal new insights into the physiology of phytopathogen-host interactions.

## Introduction

The Gram-negative bacterium *Xylella fastidiosa* is a phytopathogen that causes diseases in many economically important crops worldwide, including citrus, grapevine, plum, almond, peach, coffee (Hopkins and Purcell, 2002) and, more recently, olives (Saponari et al., 2013). In Brazil, it is the causal agent of citrus variegated chlorosis (CVC), a disease that has caused significant economic damage to the Brazilian citrus industry (Bové and Ayres, 2007). *X. fastidiosa* lives in the xylem vessels of infected plants and in the foregut of sharpshooters insect vector, which are responsible for the transmission of the bacterium directly to the xylem of the host plant (Almeida et al., 2014). Once in the xylem, *X. fastidiosa* multiplies and moves systemically colonizing the plant vessels forming biofilm, which is considered the main mechanism of *X. fastidiosa* pathogenicity. Besides, biofilm condition is required for *X. fastidiosa* insect acquisition from infected plants, characterizing the dual lifestyle of *X. fastidiosa* (Chatterjee et al., 2008).

*X. fastidiosa* in biofilm express specific genes associated with pathogenicity and adaptation in the plant (De Souza et al., 2003; Wang et al., 2012). Moreover, cells in biofilm have adaptive advantages in the environment, such as increased resistance against antimicrobial agents (Mah and O'Toole, 2001; Rodrigues et al., 2008; Muranaka et al., 2012). This resistance may be due to the presence of exopolymer matrices and changes in gene expression, making the bacteria difficult to control (Teitzel and Parsek, 2003; Rodrigues et al., 2008; Navarrete and De La Fuente, 2014). Furthermore, growth in biofilm favors the formation of persister cells, which are a small fraction of the bacterial population that exhibits multidrug tolerance without undergoing genetic changes (Keren et al., 2004; Lewis, 2007; Maisonneuve and Gerdes, 2014).

Bacterial toxin-antitoxin (TA) systems, which are highly expressed in persister cells, are primarily responsible for the persistence phenotype, as they induce a dormant state in the cells (Keren et al., 2004; Shah et al., 2006; Lewis, 2008; Wang and Wood, 2011). TA systems consist of a pair of genes in the same operon; one encodes a stable toxin that inhibits cell growth by disrupting an essential cellular process, and the other encodes the cognate labile antitoxin that prevents the toxicity of the system (Wang and Wood, 2011; Gerdes and Maisonneuve, 2012). In most cases, the antitoxin acts as a transcriptional repressor, regulating the expression of its own operon by binding to a palindromic sequence in the promoter region (Wang and Wood, 2011). This transcriptional autoregulation is controlled by a mechanism called conditional cooperativity, in which the relative toxin:antitoxin ratio in the cells determines the activation of the system (Gerdes and Maisonneuve, 2012). Additionally, the antitoxin is degraded by cellular proteases that are induced under stress conditions, which releases the toxin and promotes the operon transcription, resulting in growth inhibition and persister cell formation (Christensen et al., 2004; Maisonneuve and Gerdes, 2014).

When treated with an inhibitory concentration of copper, a compound widely used in agriculture to limit the spread of plant pathogenic bacteria and fungi (Voloudakis et al., 2005), a citrus-pathogenic strain of *X. fastidiosa* forms persister cells and induces the expression of 12 out of 65 TA systems, being *mqsRA* the most induced under this condition (Muranaka et al., 2012). The MqsRA system was first reported in *Escherichia coli* and shown to be involved in persister cell and biofilm formation (Wang and Wood, 2011). MqsR is a motility quorum sensing regulator that is directly associated with biofilm formation, as it is induced in biofilms (Ren et al., 2004), and its deletion decreases biofilm formation in *E. coli* and *X. fastidiosa* (González Barrios et al., 2006; Lee et al., 2014). This system was described in *E. coli* as composed by the MqsR toxin, which is an RNase (Brown et al., 2009) that cleaves mRNA at GCU sites (Yamaguchi et al., 2009) and requires the proteases Lon and ClpXP for its toxicity (Kim et al., 2010), and the MqsA antitoxin, which binds to the toxin via its N-terminal domain and to DNA via the helix-turn-helix (HTH) motif in its C-terminal domain (Brown et al., 2009). In the grape-pathogenic *X. fastidiosa* Temecula1 strain, MqsR also cleaves mRNA primarily at GCU sites, and MqsA inhibits the toxin by direct binding (Lee et al., 2014). Additionally, MqsRA is highly conserved between the citrus-pathogenic strain and the Temecula1 strain of *X. fastidiosa*, with approximately, 99% of amino acid sequence identity (Lee et al., 2014).

As the lifestyle (sessile or motile growth) of *X. fastidiosa* and the formation of persister cells are important for its colonization and survival (Chatterjee et al., 2008; Muranaka et al., 2012), we evaluated the function of this TA system in *X. fastidiosa* by overexpressing MqsR. We used the approach of overexpressing the toxin to study the functional role of this TA system to avoid the redundancy with other TA systems present in *X. fastidiosa*, since it was recently shown that several TA loci can be deleted without any effect on *E. coli* cells phenotype, while their overexpression strongly affect these cells (Maisonneuve et al., 2011). We verified that the toxin has regulatory functions because its overexpression induced biofilm formation and abolished the bacterial pathogenicity in citrus. In addition, overexpression of MqsR increased persister cell formation. This TA system in *X. fastidiosa* appears to have an autoregulation mechanism that controls more than just activation of the system only under stress conditions, allowing the toxin and antitoxin to be expressed in the most beneficial ratio in the cell. The results shown in this work indicate that the MqsRA TA system has a key role in adaptation and survival of *X. fastidiosa*, which directly affects its interaction with the host plant.

## Materials and methods

### Bacterial strains and growth conditions

The bacterial strains and plasmids used in this study are listed in Table S1. For the construct, we used the pXF20 vector (Lee et al., 2010), which when empty does not interfere with the bacterial fitness (Burbank and Stenger, 2016). The construct was obtained by cloning the *mqsR* open reading frame (ORF) (XF2490; http://www.lbi.ic.unicamp.br/xf/) under the control of its native promoter into the pXF20 vector. We maintained *mqsR* under control of its native promoter to verify its natural activation in the cells and under copper treatment. In addition, this strategy avoids a possible toxic effect of a high expression of MqsR driven by a constitutive promoter.

The *X. fastidiosa* strain 11399 (Coletta-Filho et al., 2001) was transformed with pXF20-*mqsR* by electroporation (1.8 kV, 200 Ω, 25 μF). The transformant cells were selected on PWG plates containing 50 μg/mL of kanamycin, and the transformation was confirmed by PCR using a specific pair of primers to detect the pXF20-*mqsR* construct (Figure S5). The primers used in these processes are shown in Table S2. Both 11399-WT and 11399-*mqsR* cells were routinely grown on PWG plates (Davis et al., 1981) at 28°C for 7 days. *E. coli* strains were grown on LB (Luria-Bertani) media (Bertani, 1951) at 37°C.

### MqsR and MqsA co-expression, co-purification and western blot

MqsR and MqsA were cloned (Table S2) and co-expressed in *E. coli* Rosetta(DE3) (Novagen) cells by cloning their ORFs in the pETDuet-1 (Novagen) expression vector, in which the MqsR protein has a His_(6)_-tag and the MqsA protein a S-tag. The co-expressed proteins were purified by affinity chromatography using a Ni-NTA agarose resin column (Qiagen). The purified extract was subjected to 15% SDS-PAGE and then transferred to a Hybond-C nitrocellulose membrane (Amersham) using the Multiphor II Novablot apparatus (LKB) for 1 h at 0.8 mA/cm^2^ of the membrane area. The membrane was blocked with 1% BSA and treated with primary and secondary antibodies using the SNAP i.d. 2.0 Protein Detection System (Millipore), and was developed using the Alkaline Phosphatase Substrate Solution (Millipore). For the primary antibodies, a dilution of 1/2500 was used for anti-His_(6)_-tag monoclonal antibody (Invitrogen), to detect MqsR, and a dilution of 1/1000 was used for anti-S-tag polyclonal antibody (Abcam), to detect MqsA. The anti-rabbit and anti-mouse secondary antibodies coupled to alkaline phosphatase (Sigma) were respectively used to detect the S-tag and His_(6)_-tag in a dilution of 1/5000 and 1/3000.

### Antibody production and ribonuclease assay

The *X. fastidiosa mqsR* and *mqsA* ORFs were cloned (Table S2) into pBAD-HisA and pET28a expression vectors, respectively, and expressed in *E. coli* Rosetta (DE3) cells (Novagen). The proteins were individually purified by affinity chromatography using a Ni-NTA agarose resin column (Qiagen) and sent to companies (Célula B and Rheabiotech), which generated polyclonal anti-MqsR and anti-MqsA antibodies through rabbit immunization.

The same purified MqsR and MqsA proteins were used in the Ribonuclease assay as described by Lee et al. (2014). Briefly, total RNA isolated from *X. fastidiosa* (1 μg) (RNeasy Mini Kit—Qiagen) was incubated for 10 min at room temperature with 10 μg of MqsR (toxin), 10 μg of MqsA (antitoxin), and 10 μg of both MqsR and MqsA. Products were visualized in 1% agarose gel electrophoresis. The RNA Integrity Number (RIN) of the samples was also determined using the 2100 BioAnalyzer system (Agilent Technologies).

### *mqsR* gene expression

The gene expression of *mqsR* during growth was assessed by growing 11399-WT and 11399-*mqsR* for 1, 2, 3, 5, and 7 days. The total RNA was extracted using the RNeasy Mini kit (Qiagen), and the DNA was eliminated using an on-column RNase-Free DNase set (Qiagen). A total of 500 ng of purified RNA from each condition was used for the synthesis of cDNA using the Reverse Transcription System kit (Promega). The real time RT-PCR was performed using the GoTaq qPCR Master Mix (Promega) in an ABI PRISM 7100 Sequence Detection System (Applied Biosystems). The *X. fastidiosa* 16S ribosomal RNA was used as endogenous control to normalize gene expression. A melting curve was conducted at the end of each run to ensure that the threshold cycle (C_T_) values obtained were from a single PCR product. The relative expression quantification (RQ) was calculated from the C_T_ values as follows (Livak and Schmittgen, 2001): dC_T_ = C_T_(target gene) − C_T_(endogenous control); ddC_T_ = dCT (treatment) − dCT (reference); RQ = 2^(−ddCT)^. The average of the RQ values from two independent experiments (two technical replicates) obtained from 11399-*mqsR* (treatment) and 11399-WT (reference) were used for statistical analysis (Student's *t*-test) to determine the significant differences between *mqsR* expression in 11399-*mqsR* in relation to 11399-WT. All qPCR primers used in this study (Table S2) were designed using the Primer Express software version 2.0 (Applied Biosystems).

### Growth curves of 11399-WT and 11399-*mqsR*

The 11399-WT and 11399-*mqsR* cells were collected from PWG plates and suspended in PBS buffer to an OD_600_ of 0.1 (approximately 10^6^ CFU/mL), and 4 mL of each cell suspension was inoculated separately in 36 mL of PW broth. Incubation was carried at 28°C with shaking at 150 rpm for 7 days. After this period, both cultures had their OD_600_ standardized to 0.1, and 300 μL aliquots of each were inoculated into 2.7 mL of fresh PW broth in polystyrene tubes. In total, 10 tubes were inoculated for each strain, which were grown at 28°C for 10 days at 150 rpm. The cell viability was measured every 24 h by 10-fold serial dilution and plating on PWG. The plates were grown at 28°C for 30 days, and the colonies were counted to determine colony formation units (CFU/mL). Additionally, a serial dilution was performed with an aliquot of the initial inoculum of both strains to determine the initial population of bacteria.

### Biofilm formation

The 11399-WT and 11399-*mqsR* cells were collected from PWG plates and suspended in PBS buffer to an OD_600_ of 0.6 (approximately 5 × 10^7^ CFU/mL), and 200 μL aliquots of each culture were inoculated into 1.8 mL of fresh PW broth in three different 24-well polystyrene culture plates (Corning). The plates were incubated at 28°C without shaking for 7, 10, and 15 days with three independent biological replicates. Each strain was inoculated in 9 wells of each plate, with 2 wells inoculated with PBS buffer as the blank sample for absorbance measurements. After these time points, the medium was discarded, and the wells were gently washed once with MilliQ water to remove planktonic and loosely adhered cells. The adhered biofilm was quantified by staining the cells with 1% crystal violet solution for 30 min at room temperature. The excess crystal violet was removed by washing the wells three times with MilliQ water. Crystal violet bound to the adhered cells was solubilized in 1 mL of 100% ethanol and quantified by measuring absorbance at 570 nm.

### EPS quantification

The 11399-WT and 11399-*mqsR* cells were collected from PWG plates and suspended in PBS buffer to an OD_600_ of 0.6. Cells from each strain were plated separately on three PWG plates. The plates were incubated at 28°C for 10 days, and the cells were resuspended in 1 mL of distilled water, standardized to an OD_600_ of 1.0, and used for EPS quantification by determining total carbohydrate concentration (Ionescu and Belkin, 2009). Briefly, 500 μL of each homogenized cell suspension was extracted by phase separation with the addition of 800 μL of chloroform. An aliquot of 400 μL of the upper phase was mixed with 800 μL of anthrone solution (Sigma) [stock solution of 1 mg/mL of anthrone reagent in concentrated H_2_SO_4_ (95–98% wt/vol)]. The mixture was incubated at room temperature for 10 min in glass tubes, and the OD_630_ of the samples was determined. The EPS content was calculated as glucose equivalents from a glucose (Sigma) standard curve (*R*^2^ = 0.99126).

### Cell aggregation

The 11399-WT and 11399-*mqsR* cells were collected from PWG plates and suspended in 2 mL of PBS buffer to an OD_600_ of 0.6, which was considered time 0. Then, without disturbing the cell suspension, an aliquot of 100 μL of the supernatant of each suspension was taken every hour for 6 h to measure the OD_600_ of the samples. The OD_600_ of the supernatant is lower with increased aggregation because cell aggregates sediment faster at the bottom of the tube.

Additionally, microscopy analysis of the aggregates was carried to 11399-WT and 11399-mqsR cells. In brief, cells were collected as above and after 1 h, without disturbing the cell suspension, aliquots of 100 μL of the supernatant and the sedimented cells of each suspension were taken for analysis. Each aliquot was stained with 100 μL of SYTO-9 5 μM (Invitrogen) for 15 min in the dark. The cells were visualized by fluorescence microscopy using a BX6 microscope (Olympus).

### Colony morphology

The 11399-WT and 11399-*mqsR* cells were collected from PWG plates and suspended in PBS buffer to an OD_600_ of 0.6. An aliquot of 100 μL of each strain was diluted from 10^−1^ to 10^−7^ by 10-fold serial dilution and plated on PWG plates containing two different BSA concentrations, 1.8 g/L and 3 g/L. We added two different concentrations of BSA to the medium to verify if indeed the morphology found in our work was related to twitching motility, since it has been demonstrated that BSA decreases twitching in *X. fastidiosa* (Galvani et al., 2007). The plates were grown at 28°C for 30 days, and the colonies were observed using a stereomicroscope.

### Gene expression of key genes related to biofilm formation and cell movement

The 11399-WT and 11399-*mqsR* cells were grown for 7 days on PWG plates and were subjected to RNA extraction, cDNA synthesis and gene expression analysis as described previously. *gltT* (glutamate symport protein) was used as endogenous control to normalize gene expression. The evaluated genes were *pilP, pilS, pilA* (associated with the biogenesis of type IV pili, which are related to twitching motility) (Li et al., 2007), *gumB* (involved in EPS polymerization and export out of the cell) (Katzen et al., 1998), *fimA* (type I fibrillin involved in cell aggregation) (Meng et al., 2005) and *eal* [responsible for the degradation of the internal messenger 3′,5′-cyclic diguanylic acid (c-di-GMP), which induces biofilm formation in *X. fastidiosa*] (Chatterjee et al., 2010; De Souza et al., 2013). The average of the RQ values from two independent experiments (two technical replicates) obtained from 11399-*mqsR* (treatment) and 11399-WT (reference) were used for statistical analysis (Student's *t*-test) to determine the significant differences between gene expression in 11399-*mqsR* in relation to 11399-WT.

### Pathogenicity assay

The 11399-WT and 11399-*mqsR* cells were collected from PWG plates and standardized to a concentration of 10^8^ CFU/mL. A volume of 10 μL of each was inoculated, separately, into 5 different sweet orange cv. Pera plants, using an entomological needle (Niza et al., 2015). The plants were kept in a greenhouse and were evaluated for the presence of CVC symptoms after 18 months of inoculation. The inoculated plants were screened for *X. fastidiosa*-infected plants by extracting the DNA from the petiole of the first leaf next to the inoculation point using the CTAB method (Murray and Thompson, 1980) and performing PCR with a specific pair of RST 31/33 primers to detect *X. fastidiosa* (Minsavage et al., 1994) (Figure S2A; Table S2). DNA samples from positive plants inoculated with 11399-*mqsR* were also analyzed by PCR using a specific pair of primers to detect the pXF20-*mqsR* construct to ensure that *X. fastidiosa* carried this construction *in planta* (Figure S2B; Table S2).

### Cell survival assay

The 11399-WT and 11399-*mqsR* cells were collected from PWG plates, suspended in PBS buffer to an OD_600_ of 0.1, and grown for 7 days in PW broth as described above. Both cultures had their OD_600_ standardized to 0.1, and 4 mL aliquots of each were inoculated into 36 mL of fresh PW broth in Erlenmeyer flasks. In total, 8 flasks were inoculated for each strain, which were grown at 28°C for 15 days at 150 rpm. Cells were exposed to 0, 1, 3 or 7 mM CuSO_4_ for 24 h (Rodrigues et al., 2008) and were collected and washed three times by centrifugation at 8000 g for 5 min at 4°C with 1 mL of PBS buffer. Cells from each strain were resuspended in 1 mL of PBS buffer, serially diluted and plated on PWG. The plates were grown at 28°C for 30 days, and the colonies were counted to determine cell survival by CFU/mL. The growth of the bacterium in the control without copper was considered 100%, and the number of colonies formed after treatment with copper was used to calculate the percentage of cell survival for each strain (Kim and Wood, 2010). The average of the cell survival values from three independent experiments obtained for 11399-*mqsR* and 11399-WT were used for statistical analysis (Student's *t*-test) to determine the significant differences (*P* < 0.05).

### Cell elongation assay

The 11399-WT and 11399-*mqsR* cells were grown for 7 days on PW broth as described previously and had their OD_600_ standardized to 0.1, and 300 μL aliquots of each sample were inoculated into 2.7 mL of fresh PW broth in glass bottom microwell (MatTek) petri dishes. In total, 8 plates were inoculated for each strain and were grown at 28°C for 15 days. The cells were exposed to 0, 1, 3, or 7 mM of copper (CuSO_4_) for 24 h, the supernatant was then discarded, and the adhered cells were gently washed once with 1 mL of PBS buffer. The adhered cells were stained using the LIVE/DEAD BacLight Bacterial Viability kit (Invitrogen) (240 μL of SYTO-9 5 μM + 6 μL of propidium iodide 20 μM) and visualized by fluorescence microscopy using a BX6 microscope (Olympus). The cell lengths were calculated using ImageJ software (http://rsbweb.nih.gov/ij/) to determine the proportion of elongated cells among the longest cells of 11399-*mqsR* and 11399-WT (>2.0 μm). Only cells longer than 4.0 μm were considered elongated because the length of normal *X. fastidiosa* cells ranges from 0.9 to 4.0 μm (Almeida et al., 2014).

### *mqsR* and *mqsA* gene expression under copper stress

The 11399-WT and 11399-*mqsR* cells were grown and treated with copper as described in the persister assay. The cells were collected by centrifugation (8000 g for 5 min at 4°C) and washed with DEPC water to remove the excess culture medium. The RNA extraction, cDNA synthesis and gene expression evaluation by qPCR were performed as described before, using the 16S ribosomal RNA as an endogenous control to normalize gene expression. The primers used in this experiment are shown in Table S2.

### MqsR and MqsA protein expression under copper stress

The 11399-WT and 11399-*mqsR* cells were grown and treated with copper as described above. The cells were collected, and total protein was extracted as follows: the cells were resuspended in 1 mL of protein extraction buffer (50 mM Tris-HCl pH 8.0, 25 mM NaCl, 5 mM EDTA pH 8.0, 2% Triton X-100 v/v), disrupted by the addition of 1 μL of lysozyme (100 mg/mL) and 1 μL of PMSF (100 mM), incubated for 25 min on ice, and then sonicated (three times for 10 s each). The samples were centrifuged at 12,000 rpm for 10 min at 4°C, the supernatant (soluble fraction) was transferred to another tube and the resulting pellet (insoluble fraction) was resuspended in 200 μL of the extraction buffer. Both were stored at −80°C. For standardization, the soluble fractions of the samples were quantified using Protein Assay solution (Bio-Rad; Bradford solution) comparing the results to a standard curve generated with known concentrations of BSA (*R*^2^ = 0.98816). The standardized samples were subjected to 15% SDS-PAGE and then transferred to a Hybond-C nitrocellulose membrane (Amersham) using the Multiphor II Novablot apparatus (LKB) for 1 h at 0.8 mA/cm^2^ of the membrane area. The membrane was blocked with 1% BSA and treated with primary and secondary antibodies using the SNAP i.d. 2.0 Protein Detection System (Millipore). For the primary antibodies, a dilution of 1/1000 was used for both anti-MqsR and anti-MqsA polyclonal antibodies. The appropriate secondary antibody, anti-rabbit coupled to horseradish peroxidase (Promega), was used at a dilution of 1/20,000 in combination with the Amersham ECL Western Blotting Detection Reagent (GE Healthcare) chemiluminescence substrates to detect proteins by exposure to Amersham Hyperfilm MP (GE Healthcare). The intensity of the signal of the bands of MqsR and MqsA was quantified using ImageJ software.

## Results

### The MqsR toxin inhibits *X. fastidiosa* growth

**Figure 1:**
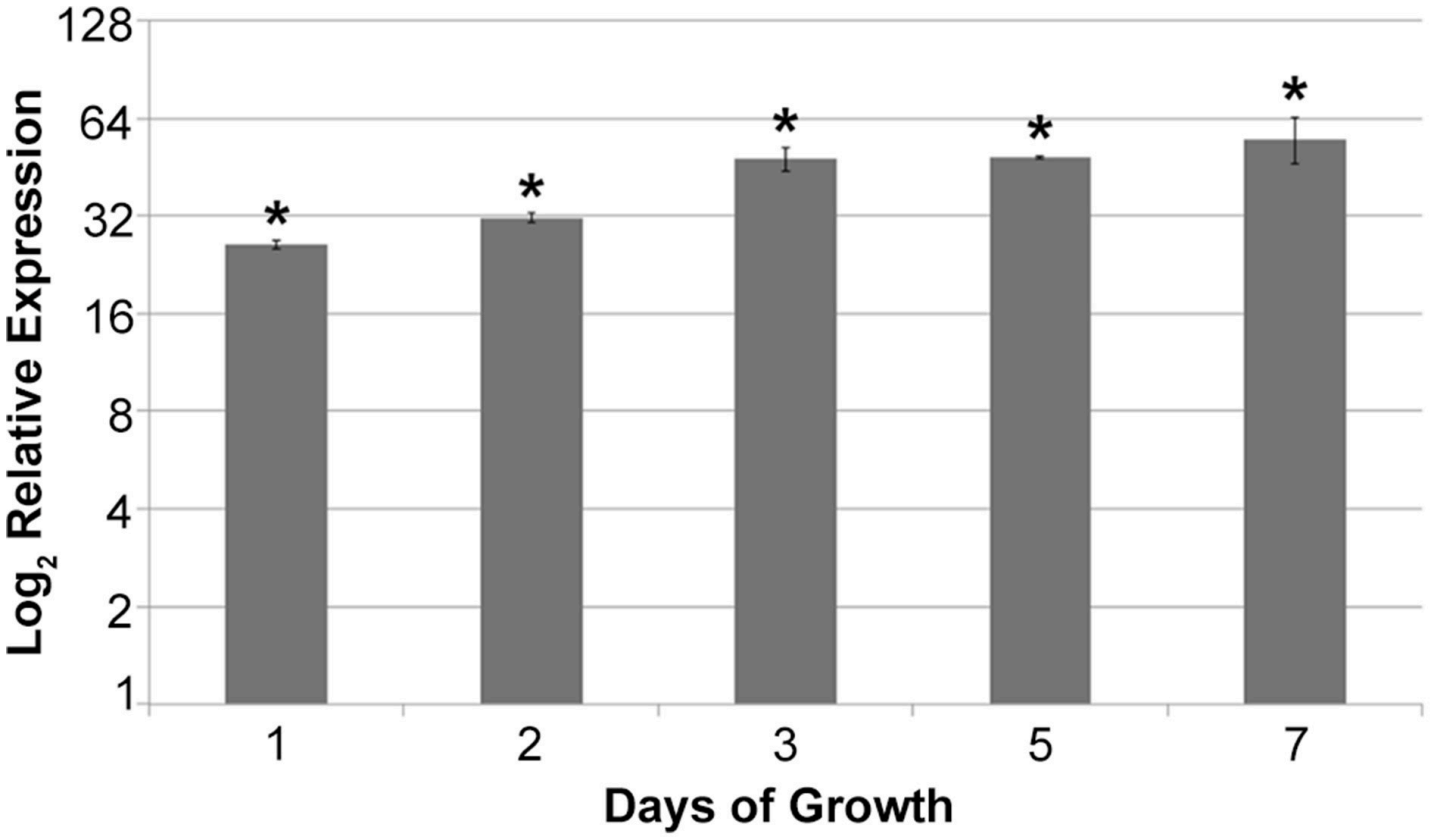
**Relative expression of *mqsR* in 11399-*mqsR* in relation to 11399-WT**. At all analyzed time points, the expression of *mqsR* was significantly higher in the transformant compared to 11399-WT. The transcript abundance was determined by real time RT-PCR. Data are shown as the mean of two independent biological replicates, and error bars indicate the standard error of the mean. ^*^Indicates significant difference between the mean values obtained for *mqsR* expression in 11399-*mqsR* compared to 11399-WT determined using Student's *t*-test (*P* < 0.05).

**Figure 2:**
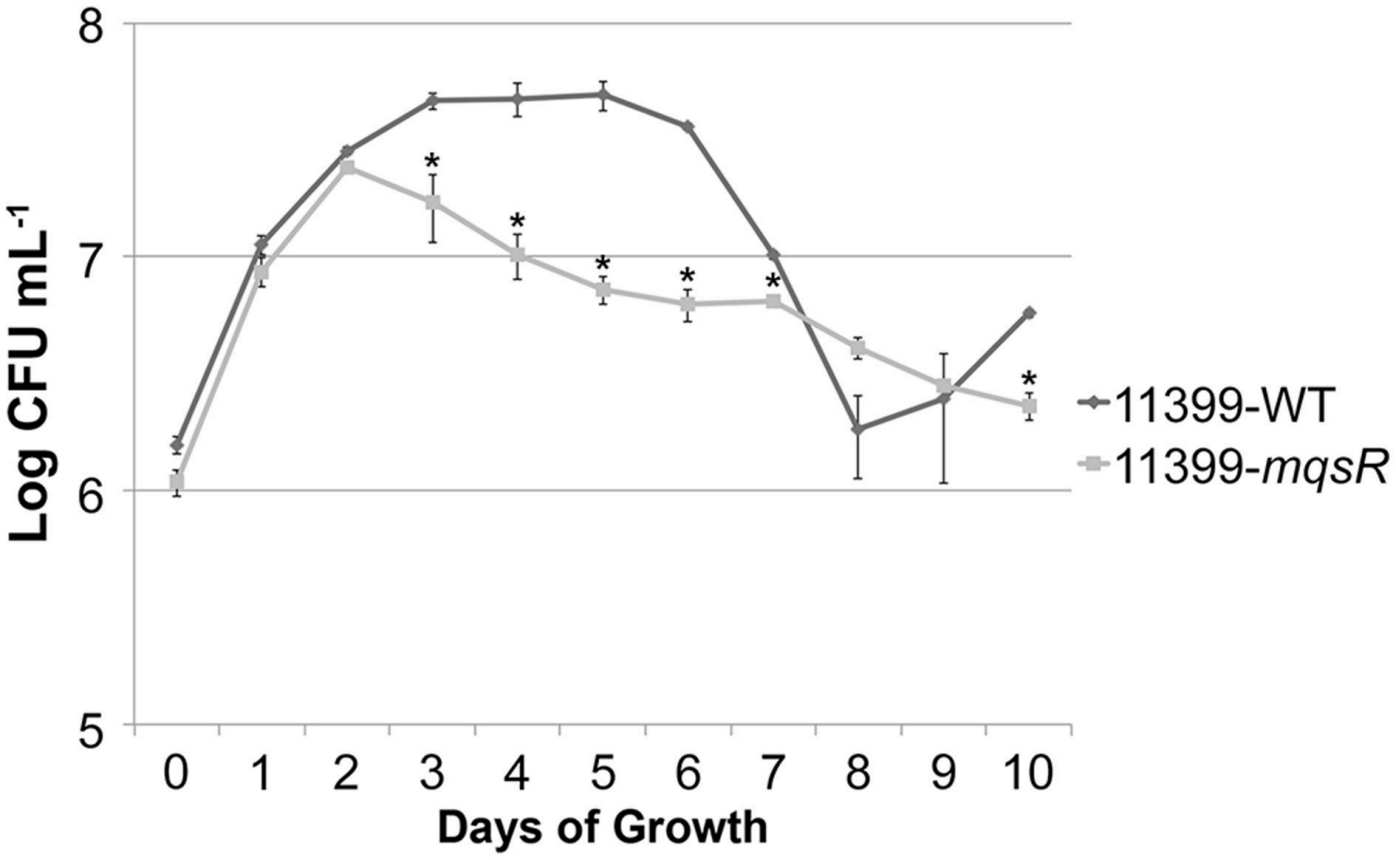
**Growth curves of 11399-*mqsR* and 11399-WT**. The 11399-*mqsR* and 11399-WT cells were standardized to an optical density (OD) at 600 nm of 0.1 and grown for 10 days in PW broth. Cell viability was analyzed every 24 h by assessing colony formation (CFU/mL). Data are shown as the mean of two independent biological replicates, and error bars indicate the standard error of the mean. There is no significant difference between 9 and 10 days of growth for 11399-WT. ^*^Indicates significant difference determined using Student's *t*-test (*P* < 0.05).

To confirm the MqsR overexpression in *X. fastidiosa*, we investigated the *mqsR* expression pattern along 7 days of growth and verified that the gene expression was significantly higher in 11399-*mqsR* than in 11399-WT at all evaluated time points (Figure 1). We also examined the effects of MqsR overexpression on *X. fastidiosa* (11399-*mqsR*) growth and observed that the toxin reduced its growth starting on the third day. The bacterial population declined without reaching the stationary phase (Figure 2). After 3 days of growth, the relative expression of *mqsR* in 11399-*mqsR*, in relation to 11399-WT, nearly doubled compared to the first 2 days of growth (Figure 1), which may explain the growth arrest observed in 11399-*mqsR* in Figure 2. We determined that the MqsRA TA system from the *X. fastidiosa* CVC strain is a functional *bona fide* TA locus because, in addition to its ability to cause growth arrest, the toxin could degrade RNA, and the antitoxin directly bound to the toxin (Figure S1).

### The MqsR toxin induces biofilm formation and reduces cell movement in *X. fastidiosa*

**Figure 3:**
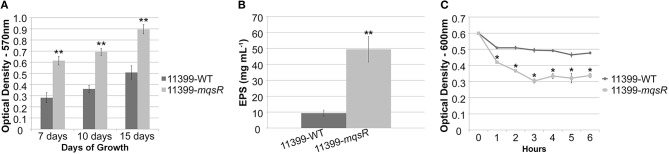
**The overexpression of MqsR induces growth in biofilm. (A)** Quantification of crystal violet staining of *X. fastidiosa* 11399-*mqsR* and 11399-WT biofilm cells attached to the bottom of polystyrene microtiter plates after 7, 10, and 15 days of growth. **(B)** EPS quantification in *X. fastidiosa* 11399-*mqsR* and 11399-WT. The EPS content was calculated as glucose equivalents from a glucose standard curve. **(C)** Cell aggregation assay of *X. fastidiosa* 11399-*mqsR* and 11399-WT. In all panels, data are shown as the mean of three independent biological replicates, and error bars indicate the standard error of the mean. Statistical significance was determined using Student's *t*-test (^*^ indicates *P* < 0.05 and ^**^ indicates *P* < 0.01).

The MqsRA TA system has a regulatory role in *E. coli* in the alternation between sessile and motile growth (Wang et al., 2011; Soo and Wood, 2013). This trait is essential for *X. fastidiosa* colonization and survival in the host plants (Chatterjee et al., 2008); therefore, we evaluated how this TA system could affect the lifestyle of *X. fastidiosa*. The overexpression of MqsR significantly induced biofilm formation in all time points, as observed in Figure 3A. We also measured the production of exopolysaccharides (EPS) because they are an essential component of biofilm formation in *X. fastidiosa* (Janissen et al., 2015). We confirmed that EPS levels were significantly higher in 11399-*mqsR* than in 11399-WT (Figure 3B). Additionally, we assessed cell aggregation, a characteristic of biofilms, and we verified that 11399-*mqsR* had a significantly more cell aggregates than 11399-WT (Figure 3C), which was also confirmed by microscopy analysis (data not shown). Overall, these results indicate that MqsR has regulatory functions and positively regulates biofilm formation in *X. fastidiosa*.

**Figure 4:**
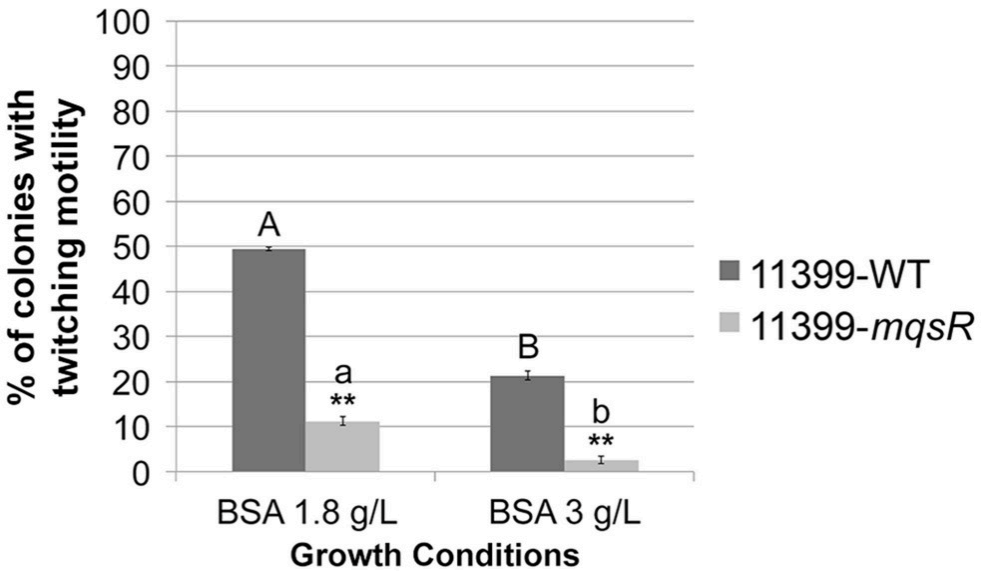
**The overexpression of MqsR reduces cell movement**. Proportion of colonies with twitching motility in 11399-*mqsR* and 11399-WT. The results are shown as the percentage of colonies with twitching motility in relation to the total bacterial population. Data are shown as the mean of three independent biological replicates, and error bars indicate the standard error of the mean. ^**^Indicates significant difference between 11399-*mqsR* and 11399-WT in both growth conditions (*P* < 0.01). Uppercase letters indicate significant difference between both growth conditions in 11399-WT (*P* < 0.01). Lowercase letters indicate significant difference between both growth conditions in 11399-*mqsR* (*P* < 0.01). Statistical significance was determined using Student's *t*-test.

To determine if MqsR could affect the colony morphology, isolated colonies were visualized under a stereomicroscope. Both 11399-*mqsR* and 11399-WT presented smooth and rough colony morphologies, but the proportion of rough colonies was different between them. It has already been reported that rough colonies are associated with twitching motility in *X. fastidiosa* (Chen et al., 2007; Li et al., 2007); and we observed a significant decrease in the twitching motility phenotype in cells overexpressing the MqsR toxin compared to 11399-WT cells in both growth conditions (Figure 4), suggesting that the transformant is less motile than the wild-type.

**Figure 5:**
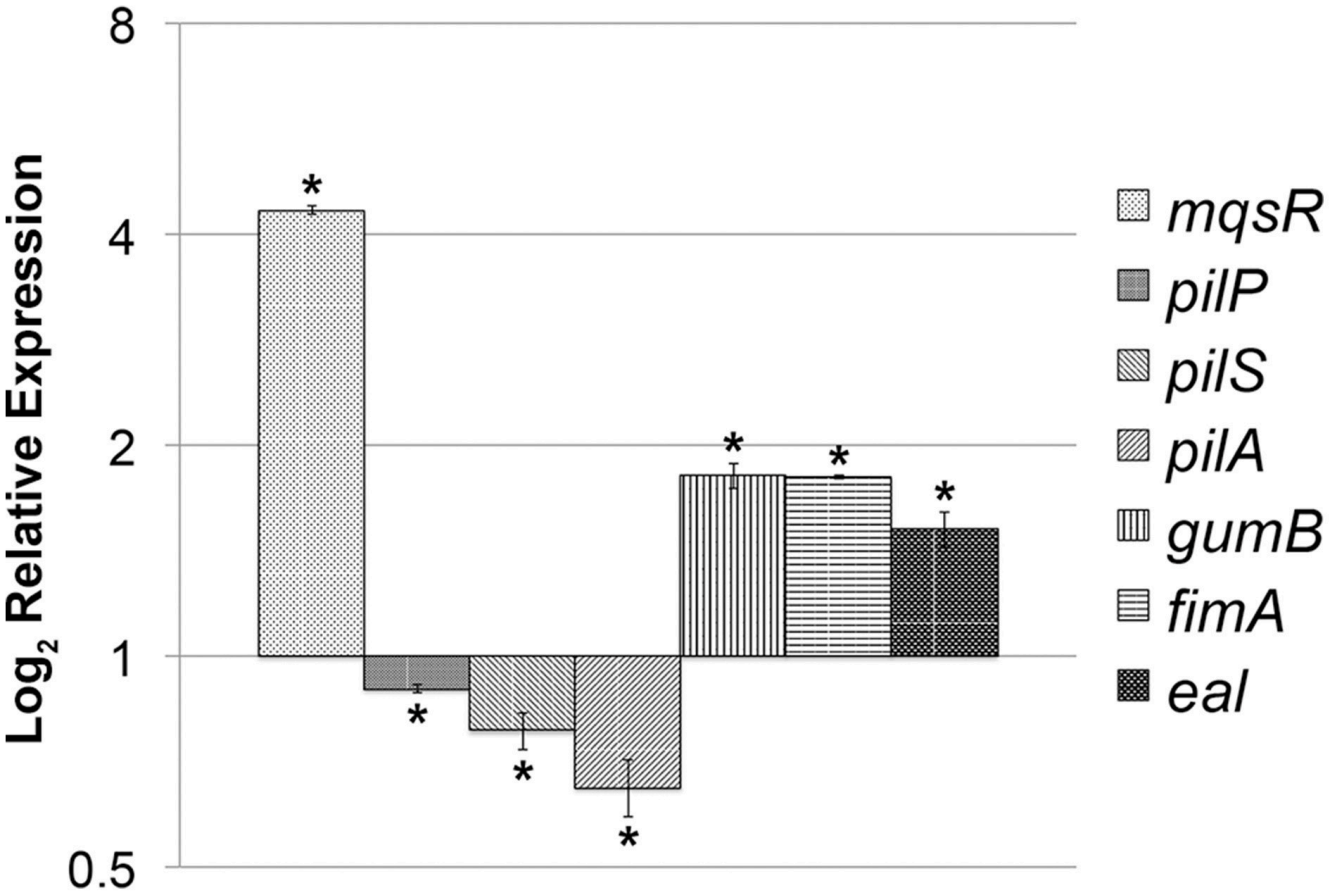
**Expression of key genes related to biofilm formation and cell movement in 11399-*mqsR***. Relative expression of the *mqsR, pilP, pilS, pilA, gumB, fimA* and *eal* genes in 11399-*mqsR* compared to 11399-WT. The transcript abundance was determined by real time RT-PCR analysis of cells after 7 days of growth on PWG plates. Data are shown as the mean of two independent biological replicates, and error bars indicate the standard error of the mean. ^*^Indicates significant difference between the mean values obtained for gene expression in 11399-*mqsR* compared to 11399-WT determined using Student's *t*-test (*P* < 0.05).

Taken together, the phenotypic results suggest that MqsR induces growth in biofilm and reduces cell movement in *X. fastidiosa* and thus has a role in bacterial lifestyle and/or fitness. In *E. coli*, the MqsRA TA system regulates these features through modulation of gene expression (Wang et al., 2011; Soo and Wood, 2013); therefore, we further determined whether overexpression of MqsR in *X. fastidiosa* could affect the expression of key genes related to biofilm formation and cell movement. There was a significant increase in the expression of *mqsR, gumB, fimA* and *eal* and a significant decrease in the expression level of *pilP, pilS*, and *pilA* in 11399-*mqsR* compared to 11399-WT (Figure 5). Thus, the overexpression of MqsR induced genes related to biofilm formation and repressed genes related to cell movement, which is consistent with the results of the phenotypic assays, suggesting that the MqsRA TA system regulates these traits through gene expression in *X. fastidiosa*.

### The overexpression of MqsR reduces *X. fastidiosa* pathogenicity *in planta*

**Figure 6:**
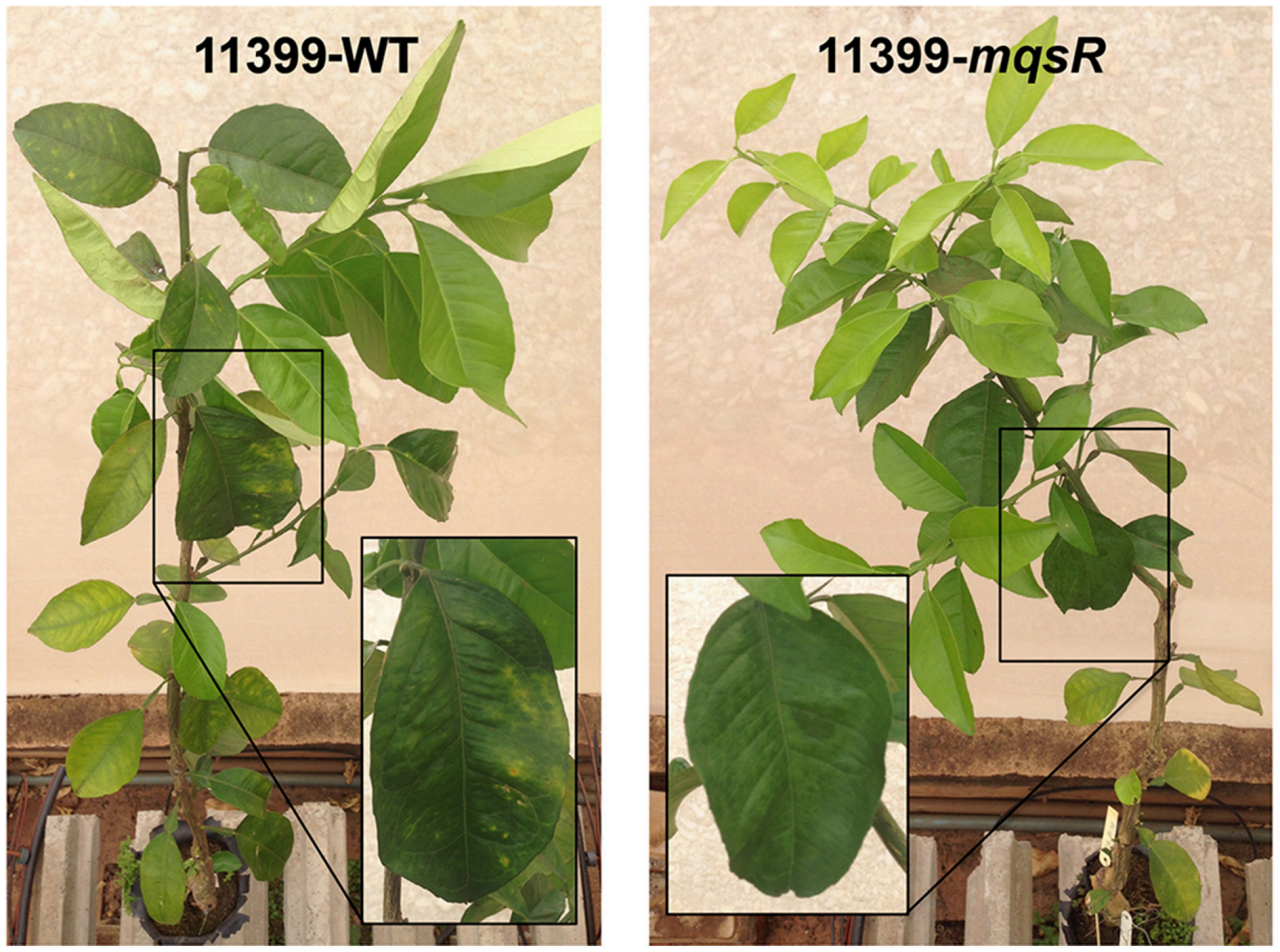
**Effect of MqsR overexpression on *X. fastidiosa* pathogenicity**. Representative pictures of sweet orange cv. Pera plants infected with 11399-WT **(left)** and 11399-*mqsR* **(right)**. The highlighted rectangles compare leaves of the same age of plants inoculated with 11399-WT or 11399-*mqsR*. CVC symptoms, such as chlorotic spots in mature leaves, appeared only in the plants inoculated with 11399-WT.

The ability of *X. fastidiosa* to colonize the host plants by moving through the xylem vessels, followed by biofilm formation, is considered to be the main cause of pathogenicity of this bacterium (Chatterjee et al., 2008). As we found that the overexpression of MqsR induced biofilm formation and reduced cell movement, we also investigated the effects of the overexpression of this toxin on *X. fastidiosa* pathogenicity. Thus, 11399-*mqsR* and 11399-WT cells were inoculated separately into sweet orange cv. Pera plants (susceptible variety) (Niza et al., 2015), and we evaluated the presence of CVC symptoms 18 months after inoculation. The overexpression of MqsR in *X. fastidiosa* decreased its pathogenicity *in planta* because CVC symptoms were verified only in plants inoculated with 11399-WT (Figure 6). We hypothesize that the overexpression of MqsR impaired the bacterial colonization in the plant because movement (Figure 4) and growth (Figure 2) of 11399-*mqsR* were reduced, with a concomitant increase in biofilm formation. We also confirmed that the 11399-*mqsR* transformant was stable in the plant (Figure S2B), with positive PCR results from plants inoculated with 11399-*mqsR* using specific primers for the pXF20-*mqsR* construct (Table S2).

### MqsR induces persister cell formation in *X. fastidiosa*

**Figure 7:**
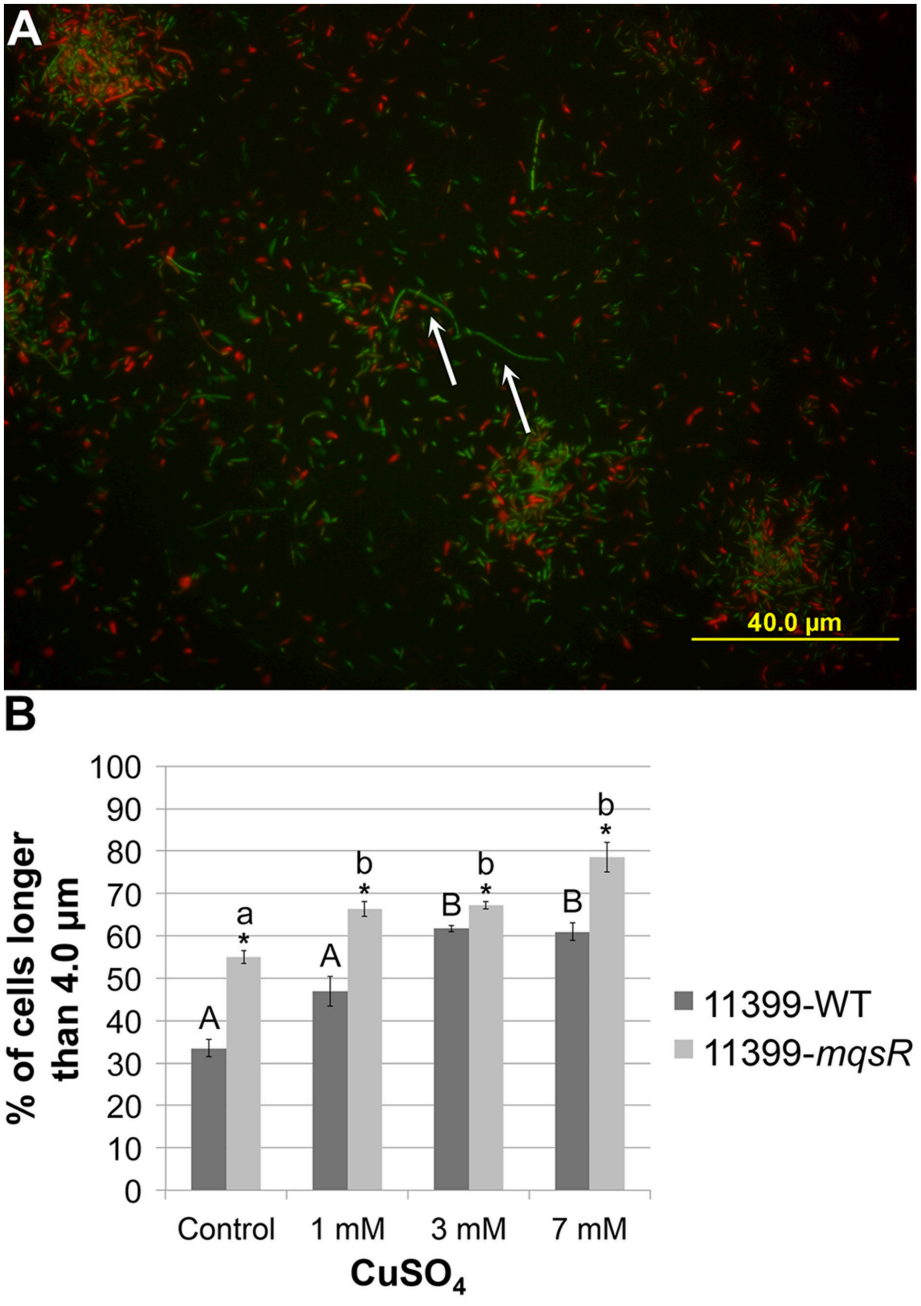
**Effect of MqsR overexpression on cell elongation**. The 11399-*mqsR* and 11399-WT cells were stained using the LIVE/DEAD BacLight Bacterial Viability kit (Invitrogen) and were visualized by fluorescence microscopy using a BX6 microscope (Olympus) with filters for green (SYTO-9—live cells) and red (propidium iodide—dead cells). The cell lengths were calculated using ImageJ software. **(A)** Representative picture of *X. fastidiosa* cells stained with SYTO-9 (green—live cells) and propidium iodide (red—dead cells). The white arrows indicate elongated cells (longer than 4.0 μm) in the picture. **(B)** Proportion of elongated cells among the longest cells in 11399-*mqsR* and 11399-WT following treatment with different concentrations of copper. Data are shown as the mean of two independent biological replicates with 200 measured cells each. The error bars indicate the standard error of the mean. ^*^Indicates significant difference between 11399-*mqsR* and 11399-WT (*P* < 0.05). Uppercase letters indicate significant difference of copper treatments compared to the control without copper in 11399-WT (*P* < 0.05). Lowercase letters indicate significant difference of copper treatments compared to the control without copper in 11399-*mqsR* (*P* < 0.05). Statistical significance was determined using Student's *t*-test.

In addition to the effects on phenotypic behavior of *X. fastidiosa*, we also investigated the role of the MqsRA TA system in persister cell formation, since, in *E. coli*, MqsR production increases persistence, and *mqsR* deletion reduces persistence (Kim and Wood, 2010). Thus, 11399-*mqsR* and 11399-WT cells were grown in PW broth, treated with 0, 1, 3, or 7 mM of copper for 24 h and plated on PW to evaluate cell survival. Cells survived only with 1 mM of copper. Under this condition, the overexpression of MqsR significantly increased cell survival by approximately 3-fold more than 11399-WT, from 3.97% ± 0.77 to 12.67% ± 1.7. As 11399-WT or 11399-*mqsR* cells did not survive the treatments with 3 and 7 mM of copper, we investigated persister cell formation in these conditions by quantifying elongated cells because the overexpression of MqsR induces this feature in *E. coli* (Kasari et al., 2010; Hong et al., 2012). Elongated cells are an indication of persister cell formation; as they represent a decrease in bacterial cell metabolism because they do not divide in this state, leading to elongation (Balaban et al., 2004; Maisonneuve et al., 2013). We evaluated the proportion of elongated cells among the longest cells of 11399-*mqsR* and 11399-WT following treatment with the different concentrations of copper (0, 1, 3, or 7 mM). Although the elongated cells represented a small fraction of the copper-treated cells (Figure 7A), the 11399-*mqsR* cells had a significantly higher proportion of elongated cells than 11399-WT in all conditions (Figure 7B), indicating that MqsR induces cell elongation and, possibly, higher persister cell formation. The copper stress also induced cell elongation because the proportion of elongated cells in 11399-*mqsR* and 11399-WT significantly increased with the increase in copper concentration compared to the controls without copper (Figure 7B). Together, the results show that the MqsRA TA system has an important role in survival during stress conditions and may increase persister cell formation in *X. fastidiosa*.

### MqsR and MqsA mRNA and protein expression in *X. fastidiosa* under copper stress

For the formation of persister cells during stress, the antitoxin needs to be degraded by cellular proteases so the toxin can be released in the cell, and the TA system can be activated (Christensen et al., 2004; Maisonneuve and Gerdes, 2014). We therefore investigated the mRNA and protein expression of MqsR and MqsA in 11399-*mqsR* and 11399-WT with different copper concentrations associated with persister cell formation.

**Figure 8:**
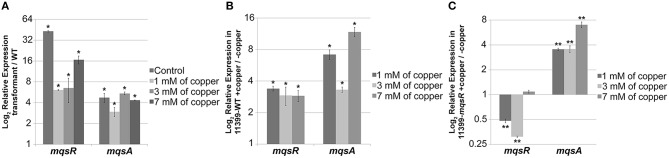
***mqsR* and *mqsA* gene expression under copper stress**. The 11399-*mqsR* and 11399-WT cells were grown for 15 days in PW broth and then exposed to 0, 1, 3, or 7 mM of copper for 24 h. The transcript abundance was determined by real time RT-PCR. **(A)** Relative expression of *mqsR* and *mqsA* in 11399-*mqsR* compared to 11399-WT after treatment with different concentrations of copper. **(B)** Relative expression of *mqsR* and *mqsA* in 11399-WT following treatment with different concentrations of copper compared to the control without copper. **(C)** Relative expression of *mqsR* and *mqsA* in 11399-*mqsR* following treatment with different concentrations of copper compared to the control without copper. In all panels, data are shown as the mean of three independent biological replicates, and error bars indicate the standard error of the mean. Statistical significance was determined using Student's *t*-test (^*^ indicates *P* < 0.05 and ^**^ indicates *P* < 0.01).

The *mqsR* and *mqsA* expression was significantly higher in 11399-*mqsR* than in 11399-WT in all treatments with copper (Figure 8A). Moreover, we also investigated how different concentrations of copper affect the expression of *mqsRA* separately in 11399-*mqsR* and 11399-WT by evaluating the expression of *mqsR* and *mqsA* in both bacteria following treatment with copper compared to the control without copper (Figures 8B,C). In 11399-WT, the expression of both *mqsR* and *mqsA* was significantly induced in all treatments with copper (Figure 8B). In 11399-*mqsR*, the expression of *mqsR* was significantly repressed when the cells were treated with 1 and 3 mM of copper, with no change in gene expression following treatment with 7 mM of copper (Figure 8C). The expression of *mqsA* was also significantly induced in 11399-*mqsR* in all evaluated conditions (Figure 8C).

**Figure 9:**
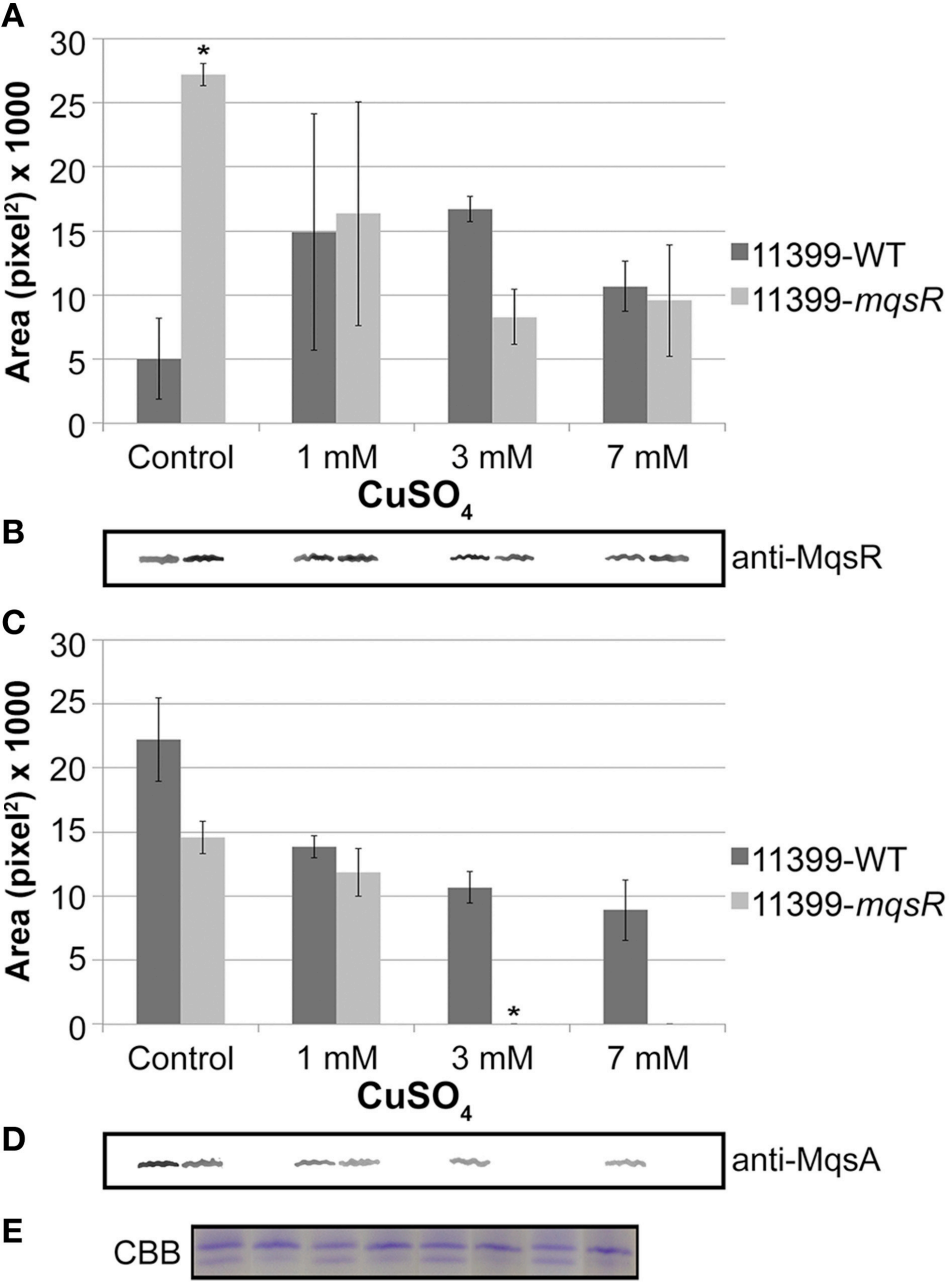
**MqsR and MqsA protein expression under copper stress**. Total protein was isolated from 11399-*mqsR* and 11399-WT cells exposed to 0, 1, 3, or 7 mM of copper, normalized by Bradford assays and evaluated by Western blotting using **(A,B)** anti-MqsR and **(C,D)** anti-MqsA polyclonal antibodies. The bands were quantified using ImageJ software. **(A)** Quantification of MqsR bands in 11399-WT and 11399-*mqsR* with different concentrations of copper. **(B)** Representative bands of MqsR generated after Western blotting with anti-MqsR. **(C)** Quantification of MqsA bands in 11399-WT and 11399-*mqsR* after treatment with different concentrations of copper. **(D)** Representative bands of MqsA generated after Western blotting with anti-MqsA. **(E)** Coomassie Brilliant Blue stain of SDS-PAGE as a loading control to show the standardization of the protein concentration in the soluble fractions of 11399-WT and 11399-*mqsR*. In panels A and C, data are shown as the mean of two independent biological replicates, and error bars indicate the standard error of the mean. ^*^Indicates significant difference between 11399-*mqsR* and 11399-WT determined using Student's *t*-test (*P* < 0.05).

Regarding the cytoplasmic levels of MqsR/MqsA, protein analysis revealed that the expression of MqsR was significantly higher in 11399-*mqsR* compared to 11399-WT without copper (Figures 9A,B). In copper-treated cells, the expression of the toxin was repressed in 11399-*mqsR*, while it was induced in 11399-WT (Figure 9A). In general, the copper stress induced the MqsR expression in 11399-WT and repressed expression in 11399-*mqsR* (Figure 9A). The MqsA level was successively repressed in both 11399-*mqsR* and 11399-WT when the copper concentration was increased, while it was absent in 11399-*mqsR* treated with 3 and 7 mM copper (Figures 9C,D). Overall, the copper stress decreased the amount of MqsA in both 11399-WT and 11399-*mqsR* (Figure 9C).

## Discussion

The formation of persister cells appears to be the main physiological role of TA systems (Wang and Wood, 2011). However, the MqsRA TA system, in addition to being the first TA system directly related to persister cell formation (Kim and Wood, 2010), was also shown to be involved in biofilm formation in *E. coli*, regulating the lifestyle of this bacterium (González Barrios et al., 2006; Kasari et al., 2010; Wang et al., 2011; Soo and Wood, 2013). In this study, we showed that the MqsR toxin is also capable of inducing biofilm formation (Figure 3) and repressing cell movement in *X. fastidiosa* (Figure 4). Additionally, it was also shown to be involved in persister cell formation (Figure 7). The formation of biofilm and cell movement are opposing processes in this bacterium (Chatterjee et al., 2008); thus, we demonstrated that the MqsRA TA system also plays a key role in alternation between sessile and motile growth in *X. fastidiosa*.

Consistent with our results, it was reported in the Temecula1 strain of *X. fastidiosa* that the deletion of *mqsR* reduces biofilm formation, and the deletion of *mqsA* induces biofilm formation, indicating that excess of free toxin in the cell creates a favorable environment for the development of biofilm (Lee et al., 2014). Because biofilms have increased resistance to antimicrobial agents (Mah and O'Toole, 2001; Rodrigues et al., 2008; Muranaka et al., 2012), the free toxin in the cell may increase in response to stress, and the induction of biofilm formation would be a defense mechanism. Nonetheless, to determine if the MqsR toxin could also contribute to the formation of biofilm in the absence of stress, we evaluated the expression of *mqsR* in 11399-WT during 7 days of growth and observed that the *mqsR* expression is higher in the first days of *X. fastidiosa* growth (Figure S3). This suggests that the MqsRA TA system may also have a role in the initial growth of this bacterium and possibly regulates biofilm formation. In *X. fastidiosa*, the small diffusible signaling molecule called DSF (diffusible signaling factor), which is synthesized by the RpfF protein, mediates cell-to-cell quorum sensing signaling, inducing biofilm formation when the cells are at a high density (Chatterjee et al., 2008). Interestingly, in an *rpfF*-deleted *X. fastidiosa* strain the *mqsR* toxin gene showed high expression (Wang et al., 2012). Thus, MqsR is likely a regulator that controls the population dynamics of cells in the absence of DSF in *X. fastidiosa*, as it occurs during the first days of bacterial growth.

In *E. coli*, MqsA binds to the promoter region of specific genes in addition to its own promoter and represses their expression (Brown et al., 2009; Kim et al., 2010; Wang et al., 2011; Soo and Wood, 2013). At its own promoter, the antitoxin binds to the DNA via the Asn^97^ and Arg^101^ residues of its HTH motif in two different palindromic sequences: 5′-ACCT (N)_3_ AGGT and 5′ -TAACCT (N)_3_ AGGTTA (Yamaguchi et al., 2009; Brown et al., 2011). When bound to the promoter regions of *rpoS* (sigma factor S) and *csgD*, the antitoxin represses the production of c-di-GMP and curli and cellulose, leading to biofilm repression in *E. coli* (Wang et al., 2011; Soo and Wood, 2013). Hence, MqsA is considered a biofilm formation inhibitor (Wang et al., 2011), and MqsR acts as a transcriptional de-repressor, activating the genes repressed by the antitoxin and promoting biofilm formation (Brown et al., 2013). In *X. fastidiosa*, MqsA has the same Asn^97^ and Arg^101^ residues in its HTH motif (data not shown) and a 5′-TAACCT (N)_3_ AAGTTA sequence in its promoter region that is very similar (91.7% of similarity—Figure S4) to the target palindromic sequence of *mqsRA* in *E. coli*. Thus, we hypothesize that gene regulation by MqsA in *X. fastidiosa* occurs in a similar manner as in *E. coli*. However, analyzing the promoter region of regulators that were induced under copper stress (a condition in which *mqsRA* was highly expressed), such as *lysR* (XF1721), *actS* (XF2577), *trpR* (XF1920), and *opdE* (XF1749) (http://www.lbi.ic.unicamp.br/xf/), as shown by Muranaka et al. (2012), either manually or using the PATLOC (Pattern Locator) online software (http://www.cmbl.uga.edu/software/patloc.html) did not reveal any possible regulatory sequences similar to the ones found in *X. fastidiosa* and *E. coli* (Figure S4). In addition, *X. fastidiosa* does not have *rpoS*, which is an important target for the antitoxin in *E. coli* (Wang et al., 2011). Analysis of the promoter region of other sigma factors that are present in the genome of *X. fastidiosa* [*rpoD* (XF1350), *rpoE* (XF2239), *rpoH* (XF2691) and *rpoN* (XF1408); http://www.lbi.ic.unicamp.br/xf/] and of the genes induced by MqsR in this work (Figure 5) also did not identify any regulatory sequences. These analyses suggest that the regulation mechanism of MqsRA in *X. fastidiosa* is possibly different from the one described for *E. coli*, with different target genes having different palindromic binding sequences. Indeed, we were able to find several genes using the PATLOC online software to search the *X. fastidiosa* genome for sequences that retain part of the probable regulatory sequence of *mqsRA* (5′-AAC (N)_7_ GTT) (data not shown) and could thus be binding sequences for MqsA in *X. fastidiosa*. Among them, 77 of these sequences were in intergenic regions, including *pilR* (XF2545), *clpP* (XF0381, XF1187), *rpoE* (XF2239), and *mqsR* (XF2490) itself (http://www.lbi.ic.unicamp.br/xf/), which we point out for having regulatory functions.

Furthermore, as MqsR is an mRNA interferase that degrades mRNA specifically at GCU sites in *X. fastidiosa* (Lee et al., 2014), it could regulate gene expression by differential mRNA decay, favoring the expression of genes related to growth in biofilm, such as the ones analyzed in this work (Figure 5). In accordance with this hypothesis, a transcriptome study with a MqsR-overexpressing *E. coli* strain showed induction of 132 transcripts, many of which were related to biofilm formation and stress response (Kim et al., 2010).

We showed that the overexpression of the toxin led to growth arrest (Figure 2) and reduced movement (Figure 4), likely a consequence of the reduced expression of type IV pili genes (Figure 5). Consequently, no disease symptoms developed in the host plant (Figure 6). In fact, it has been shown that type IV pili are essential for *X. fastidiosa* pathogenicity (Meng et al., 2005; De La Fuente et al., 2007).

Therefore, based on the phenotypes associated with MqsR function, we suggest that the levels of the TA components must be tightly controlled. Indeed, the overexpression of MqsR also led to a higher cell survival in *X. fastidiosa* under copper stress and the formation of persister cells, which was confirmed by increases in elongated cells (Figure 7). In *X. fastidiosa*, persister cells could play a role in survival under environmental stresses, allowing regrowth and consequently recolonization after the end of the stresses. This was observed in other bacterial models, in which persistence represents an important mechanism for survival in harsh conditions (Wang and Wood, 2011). The mechanism by which MqsRA induces the formation of persister cell has already been described in *E. coli*. It is known that MqsR requires the proteases Lon and ClpXP, as well as *hha* (a toxin that controls cell death and biofilm dispersal) and *cspD* (a stress-induced toxin that inhibits DNA replication) for its toxicity during persister cell formation (Yamanaka et al., 2001; García-Contreras et al., 2008; Kim and Wood, 2010; Kim et al., 2010). However, in *X. fastidiosa*, none of these genes were induced under copper stress (Muranaka et al., 2012), and this bacterium does not have *hha*, suggesting, as observed for biofilm formation, that the mechanism involved in persister cell formation via MqsRA in *X. fastidiosa* could be similar but not identical to that described in *E. coli*.

By overexpressing MqsR in *X. fastidiosa*, we determined how different concentrations of copper affected the behavior of the MqsRA TA system in this bacterium (Figures 8, 9). In 11399-WT, the copper stress activated the transcription of *mqsRA* (Figure 8B), increasing the levels of MqsR (Figure 9A) while reducing the levels of MqsA (Figure 9C), which is probably degraded by cellular proteases that are induced during stress conditions and de-repress the transcription of the system. Thus, 11399-WT responded to stress according to what is known for TA systems activation mechanism (Christensen et al., 2004; Maisonneuve and Gerdes, 2014). This transcriptional autoregulation of MqsRA occurs through the mechanism of conditional cooperativity, in which the relative toxin:antitoxin ratio determines the activation of the system in the cell. Transcription is repressed when the amount of the toxin is equal to or lower than that of the antitoxin, promoting transcriptional activation when its amount exceeds that of the antitoxin (Gerdes and Maisonneuve, 2012). Interestingly, in 11399-*mqsR*, both the gene and protein expressions of MqsR were repressed with the increase in copper concentration in Figures 8C, 9A. Considering that the toxin is under the control of its native promoter in the transformant, we expected that 11399-*mqsR* would behave similarly to 11399-WT regarding gene expression, with a higher level of toxin transcripts in the transformant (because of the additional copies of the *mqsR* gene) reflected in the protein levels. However, we observed different regulation for both the toxin and antitoxin in 11399-*mqsR* in the presence of copper, where higher levels of *mqsA* were observed in 11399-*mqsR* compared to 11399-WT in all copper concentrations (Figure 8A). On the other hand, the MqsA protein was not observed when the transformant was treated with 3 and 7 mM of copper (Figure 9C), with a concomitant reduction in MqsR (Figure 9A). We believe that 11399-*mqsR* perceives the stress and responds in a stronger manner, leading to a higher degradation of the antitoxin and, consequently, leading to the degradation of the toxin, as MqsR levels were reduced as the copper concentrations increased (Figure 9A), with a similar trend observed for transcripts (Figure 8C). We suggest that this regulation mechanism probably occurs to prevent a large imbalance in the toxin:antitoxin ratio in the cell. Because 11399-*mqsR* produced more persister cells than 11399-WT (Figure 7), the higher amount of free toxin in 11399-*mqsR* is likely beneficial for stress survival. Nevertheless, the ratio between the toxin and antitoxin in the cell needs to be fine-tuned, as this balance is important to determine the formation of persister cells, and this only occurs when the free toxin level reaches a certain threshold (Rotem et al., 2010). The amount of free toxin that exceeds this threshold, which is necessary for the formation of persister cells, is also responsible for determining the duration of the dormancy induced; thus, the more free toxin in the cell, the higher the quantity of required antitoxin for the cells to resume growth (Rotem et al., 2010; Ayrapetyan et al., 2015). Therefore, a high quantity of free toxin in the cell is not desirable, as it would result in difficulties in restarting growth, which may explain the decrease in MqsR with the increase in stress in 11399-*mqsR* (Figure 9A); concomitantly, the high level of *mqsA* transcripts present in 11399-*mqsR* (Figure 8C) could ensure the rapid production and accumulation of the antitoxin for the bacteria to restrict the activity of the toxin and facilitate the cells to resume growth. In support of our hypothesis, it was recently shown that the toxin HigB of *Caulobacter crescentus* can either promote or inhibit cell growth depending on its expression level, which is controlled by the repression of the HigBA promoter, and the type and intensity of stress (Kirkpatrick et al., 2016).

**Figure 10:**
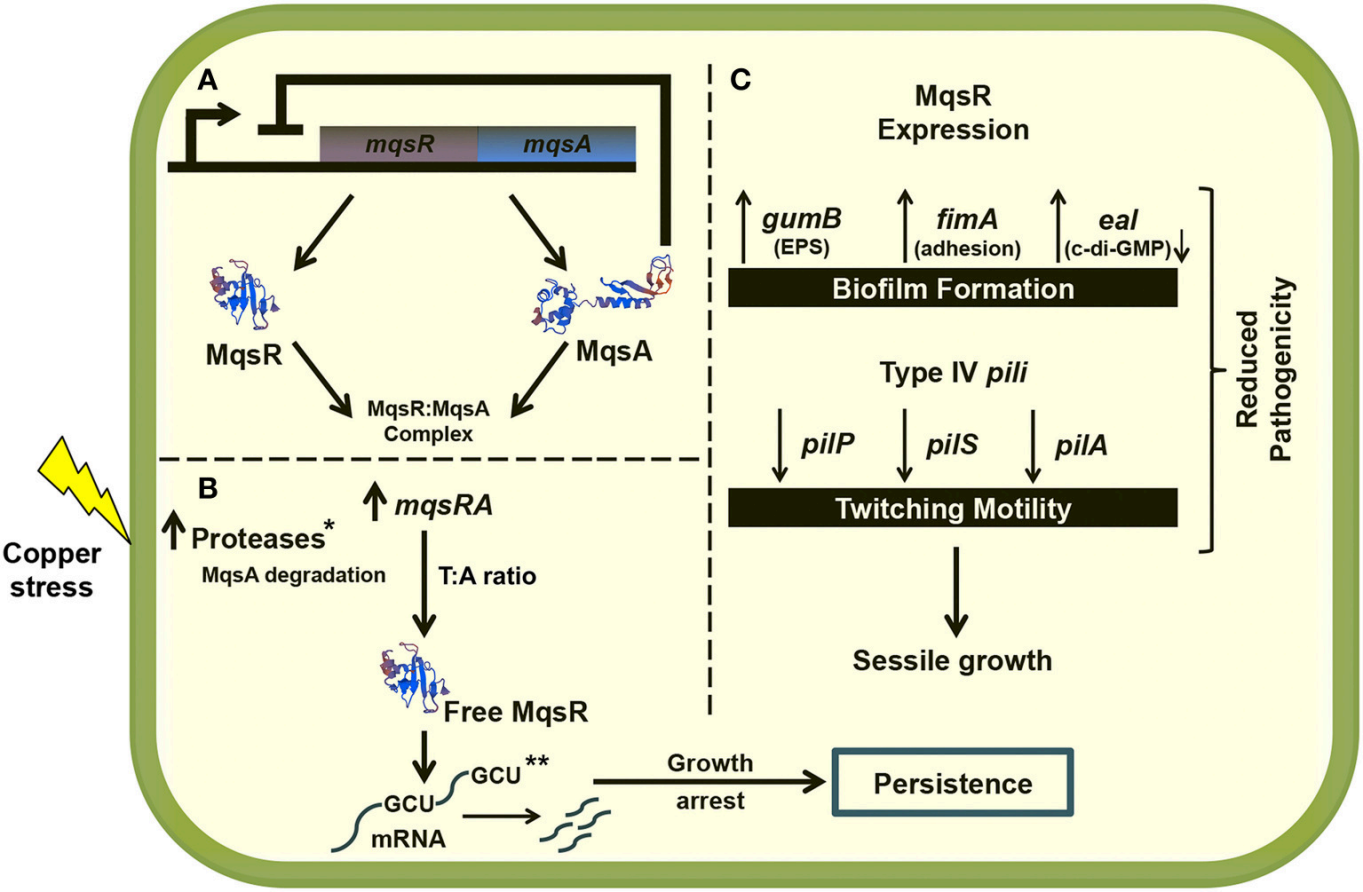
**Proposed model of MqsRA role in *X. fastidiosa* cells. (A)** Under normal conditions, the MqsA antitoxin binds to the MqsR toxin, forming the TA complex and inhibiting the toxicity of the system. In normal condition, the antitoxin also represses the transcription of *mqsRA*. **(B)** Under copper stress, the antitoxin is possibly degraded by proteases that are active under stress conditions, promoting the transcription of the TA system. The system is induced by copper stress, and it may autoregulate the expression of toxin and antitoxin in the most beneficial ratio for the cell to oppose stress, through formation of persister cells. Degradation of mRNA specifically at GCU sites by MqsR is probably responsible by the cell arrest leading to persister cell formation. **(C)** The MqsR toxin also induces the expression of genes related to biofilm formation and represses the expression of genes related to cell movement. Taken together, the data indicate that the expression of MqsR benefits the sessile growth in *X. fastidiosa*, as also observed in the phenotypic experiments, which reduces its pathogenicity in planta. In the figure, the lightning bolt indicates copper stress, → indicates induction and ⊥ indicates repression. The modeling of the proteins presented in the figure was performed using the SWISS-MODEL online software (swissmodel.expasy.org). ^*^Not identified in this work but shown for *E. coli* (Christensen et al., 2004; Maisonneuve and Gerdes, 2014). ^**^Degradation of mRNA specifically at GCU sites was demonstrated for the *X. fastidiosa* grape-pathogenic strain Temecula1 (Lee et al., 2014).

Although it is well known that persister cells are largely responsible for the recalcitrance of infectious diseases caused by bacterial biofilms in humans (Wang and Wood, 2011), the involvement of the TA systems in cell physiology and persister cells in phytopathogens has not been well-characterized. Therefore, this work will broaden the knowledge of the lifestyle and survival mechanisms of *X. fastidiosa* in biofilm in response to antimicrobial compounds. To our knowledge, this is the first time that a direct link between the MqsRA TA system and biofilm and persister cell formation in a phytopathogen has been described, and the first time that the behavior of a TA system with different concentrations of copper, a widely used antimicrobial agent in agriculture, has been assayed. Further studies with 11399-*mqsR* may elucidate the genetic mechanisms by which this TA system regulates the *X. fastidiosa* population dynamics and contributes to its fitness in the host environment. Figure 10 summarizes our understanding to date of the role of MqsRA in *X. fastidiosa* cells.

## Author contributions

MM designed and performed experiments, analyzed data and wrote the paper; BN performed experiments; MT analyzed data and revised the paper; AS designed experiments, analyzed data and revised the paper.

## Funding

This work was supported by research grants from Fundação de Amparo à Pesquisa do Estado de São Paulo (2010/50712-9, 2013/17485-7). MM is a fellow MSc. from FAPESP (2013/02014-9). AS and MT are recipients of research fellowships from CNPq.

### Conflict of interest statement

The authors declare that the research was conducted in the absence of any commercial or financial relationships that could be construed as a potential conflict of interest.

## Supplementary material

Figure S1 ***mqsRA* is a *bona fide* TA system in the CVC-causing strain of *X. fastidiosa***. The purified extract of bacteria co-expressing MqsR and MqsA was submitted to Western blot using either anti-His_(6)_-tag monoclonal antibody to detect MqsR **(A)** or anti-S-tag polyclonal antibody to detect MqsA **(B)**. Since both MqsR and MqsA were detected after purification, this means that MqsR and MqsA directly interact. M—PageRuler Prestained Protein Ladder (Thermo Scientific). 1—Purified extract from the co-expression of MqsR and MqsA. The molecular weight of MqsR is, approximately, 14.0 KDa with the His_(6)_-tag. The molecular weight of MqsA-S-tag is approximately 18.0 KDa. **(C)** Ribonuclease assay with the purified MqsR and MqsA, individually and together using total RNA isolated from *X. fastidiosa*. The figure shows that MqsR is a ribonuclease and that MqsA inhibits its action when both are added to the reaction. MqsA alone does not show any effect on the RNA. 16S and 23S ribosomal RNAs are indicated in the figure. **(D)** RNA integrity number (RIN) of the samples from **(C)**. Only the sample treated with the MqsR toxin has a low RIN that indicates RNA degradation.

Figure S2 **PCR analysis of the plants inoculated with 11399-*mqsR* and 11399-WT. (A)** PCR carried out with a specific RST 31/33 pair of primers to detect *X. fastidiosa* in DNA samples extracted from sweet orange cv. Pera plants inoculated with 11399-*mqsR* and 11399-WT. The expected size for the RST 31/33 amplicon is 733 bp. M—GeneRuler 1 kb Plus DNA Ladder (Thermo Scientific). N—DNA from healthy plant (negative control). Wells 1–5 indicate PCR reaction carried out with plants inoculated with 11399-WT, in which only plants 2, 3, and 4 were positive. Wells 6–10 indicate PCR reaction carried out with plants inoculated with 11399-*mqsR*, in which only plants 6, 7, and 8 were positive. **(B)** PCR carried out with a specific pair of primers to detect the pXF20-*mqsR* construct in DNA samples extracted from sweet orange cv. Pera plants inoculated with 11399-*mqsR*. The expected size of the amplicon is 760 bp. M—GeneRuler 1 kb Plus DNA Ladder (Thermo Scientific). Plant 2—sweet orange cv. Pera plant inoculated with 11399-WT (negative control). +—plasmid miniprep (PureYield Plasmid Miniprep System—Promega) of the pXF20-*mqsR* construct (positive control). Plants 6, 7, and 8—PCR reaction carried out with positive plants inoculated with 11399-*mqsR*, in which the presence of the amplicon of 760 bp in all of them confirms the stability of the pXF20-*mqsR* construct in planta after 18 months of inoculation.

Figure S3 **Expression of *mqsR* in 11399-WT in the first days of growth**. Relative expression of *mqsR* in 11399-WT at 1, 2, 3, and 5 days compared to 7 days of growth. The transcript abundance was determined by real time RT-PCR. Data are shown as the mean of two independent biological replicates, and error bars indicate the standard error of the mean. ^**^Indicates significant difference determined using Student's *t*-test (*P* < 0.01).

Figure S4 **Pairwise alignment of *Escherichia coli* and *Xylella fastidiosa* MqsA palindromic binding sequences**. The MqsA probable binding sequence in the *mqsRA* promoter of *X. fastidiosa* (located at position −24) shares a 91.7% similarity with palindrome 2 (located at position −43) of the *E. coli mqsRA* promoter. There is a guanine instead of an adenine (highlighted in yellow in the figure) in *X. fastidiosa* compared to *E. coli*. The (N)_3_ region of the sequences is highlighted in blue in the figure. The alignment of the sequences was performed using the Clustal Omega online software (http://www.ebi.ac.uk/Tools/msa/clustalo/).

Figure S5 **Confirmation of *X. fastidiosa* carrying pXF20-*mqsR***. PCR performed with a specific pair of primers to detect the pXF20-*mqsR* construct in DNA samples extracted from 11399-*mqsR*. The expected size of the amplicon is 760 bp. M—GeneRuler 1 kb Plus DNA Ladder (Thermo Scientific). +—plasmid miniprep (PureYield Plasmid Miniprep System—Promega) of the pXF20-*mqsR* construct (positive control). N—PCR reaction carried out with DNA extracted from 11399-WT (negative control). Wells 1 and 2 indicate PCR reaction carried out with DNA extracted from 11399-*mqsR*. Wells 3 and 4 indicate PCR reaction carried out with MilliQ water to assess possible contamination in the pair of primers. The 760 bp amplicon was only observed in the positive control and 11399-*mqsR* (wells 1 and 2), confirming the transformation of *X. fastidiosa* with the pXF20-*mqsR* construct.

Table S1 **Bacterial strains and plasmids used in this study**.

Table S2 **Oligonucleotide primers used in this study**.
