# Supplementary figures and images for: The MqsRA Toxin-Antitoxin System from Xylella fastidiosa Plays a Key Role in Bacterial Fitness, Pathogenicity, and Persister Cell Formation
Source: Front Microbiol. 2016 Jun 10;7:904. doi: 10.3389/fmicb.2016.00904 (PMC4901048; doi:10.3389/fmicb.2016.00904)

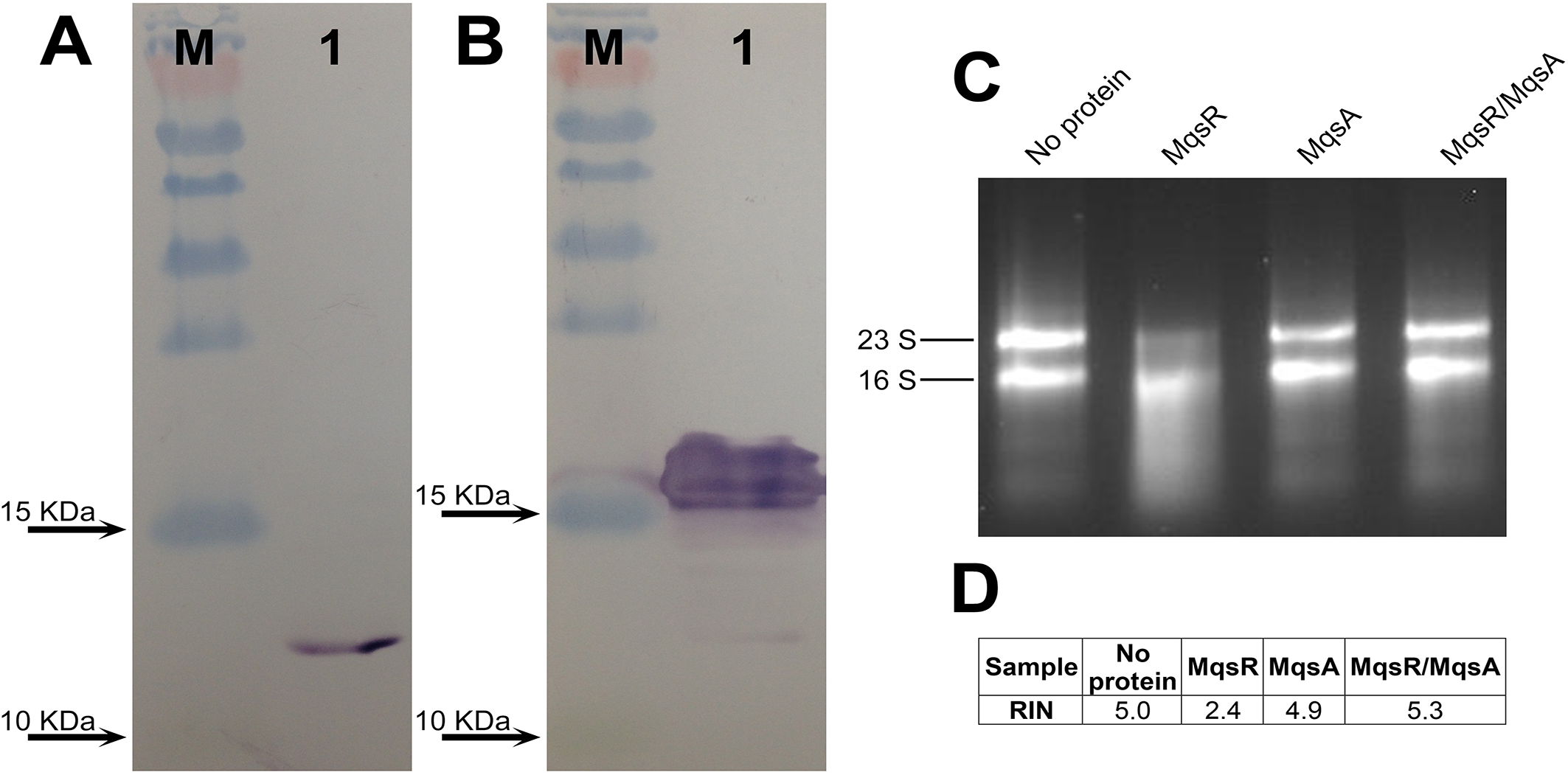

Supplement: Figure S1 — mqsRA is a bona fide TA system in the CVC-causing strain of X. fastidiosa. The purified extract of bacteria co-expressing MqsR and MqsA was submitted to Western blot using either anti-His(6)-tag monoclonal antibody to detect MqsR (A) or anti-S-tag polyclonal antibody to detect MqsA (B). Since both MqsR and MqsA were detected after purification, this means that MqsR and MqsA directly interact. M—PageRuler Prestained Protein Ladder (Thermo Scientific). 1—Purified extract from the co-expression of MqsR and MqsA. The molecular weight of MqsR is, approximately, 14.0 KDa with the His(6)-tag. The molecular weight of MqsA-S-tag is approximately 18.0 KDa. (C) Ribonuclease assay with the purified MqsR and MqsA, individually and together using total RNA isolated from X. fastidiosa. The figure shows that MqsR is a ribonuclease and that MqsA inhibits its action when both are added to the reaction. MqsA alone does not show any effect on the RNA. 16S and 23S ribosomal RNAs are indicated in the figure. (D) RNA integrity number (RIN) of the samples from (C). Only the sample treated with the MqsR toxin has a low RIN that indicates RNA degradation. [file Image1.TIF]

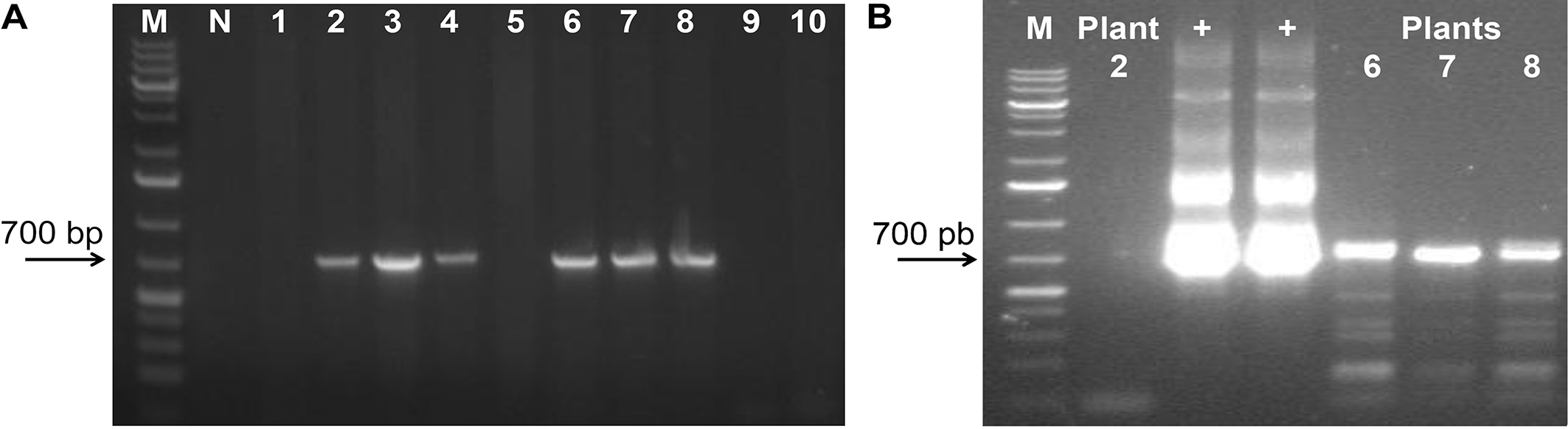

Supplement: Figure S2 — PCR analysis of the plants inoculated with 11399-mqsR and 11399-WT. (A) PCR carried out with a specific RST 31/33 pair of primers to detect X. fastidiosa in DNA samples extracted from sweet orange cv. Pera plants inoculated with 11399-mqsR and 11399-WT. The expected size for the RST 31/33 amplicon is 733 bp. M—GeneRuler 1 kb Plus DNA Ladder (Thermo Scientific). N—DNA from healthy plant (negative control). Wells 1–5 indicate PCR reaction carried out with plants inoculated with 11399-WT, in which only plants 2, 3, and 4 were positive. Wells 6–10 indicate PCR reaction carried out with plants inoculated with 11399-mqsR, in which only plants 6, 7, and 8 were positive. (B) PCR carried out with a specific pair of primers to detect the pXF20-mqsR construct in DNA samples extracted from sweet orange cv. Pera plants inoculated with 11399-mqsR. The expected size of the amplicon is 760 bp. M—GeneRuler 1 kb Plus DNA Ladder (Thermo Scientific). Plant 2—sweet orange cv. Pera plant inoculated with 11399-WT (negative control). +—plasmid miniprep (PureYield Plasmid Miniprep System—Promega) of the pXF20-mqsR construct (positive control). Plants 6, 7, and 8—PCR reaction carried out with positive plants inoculated with 11399-mqsR, in which the presence of the amplicon of 760 bp in all of them confirms the stability of the pXF20-mqsR construct in planta after 18 months of inoculation. [file Image2.TIF]

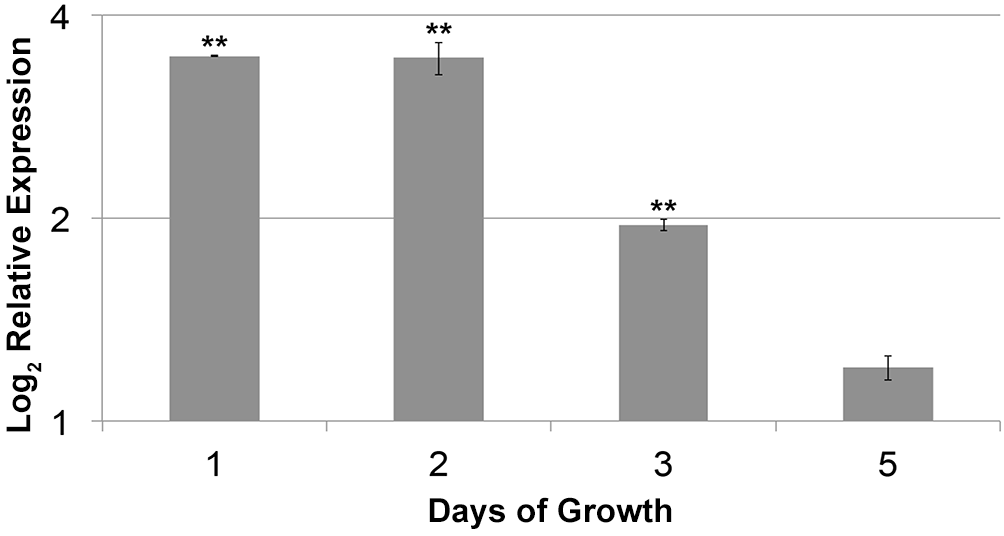

Supplement: Figure S3 — Expression of mqsR in 11399-WT in the first days of growth. Relative expression of mqsR in 11399-WT at 1, 2, 3, and 5 days compared to 7 days of growth. The transcript abundance was determined by real time RT-PCR. Data are shown as the mean of two independent biological replicates, and error bars indicate the standard error of the mean. **Indicates significant difference determined using Student's t-test (P < 0.01). [file Image3.TIF]

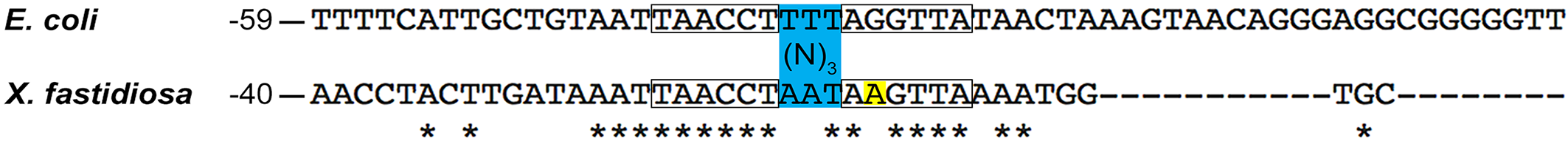

Supplement: Figure S4 — Pairwise alignment of Escherichia coli and Xylella fastidiosa MqsA palindromic binding sequences. The MqsA probable binding sequence in the mqsRA promoter of X. fastidiosa (located at position −24) shares a 91.7% similarity with palindrome 2 (located at position −43) of the E. coli mqsRA promoter. There is a guanine instead of an adenine (highlighted in yellow in the figure) in X. fastidiosa compared to E. coli. The (N)3 region of the sequences is highlighted in blue in the figure. The alignment of the sequences was performed using the Clustal Omega online software (http://www.ebi.ac.uk/Tools/msa/clustalo/). [file Image4.TIF]

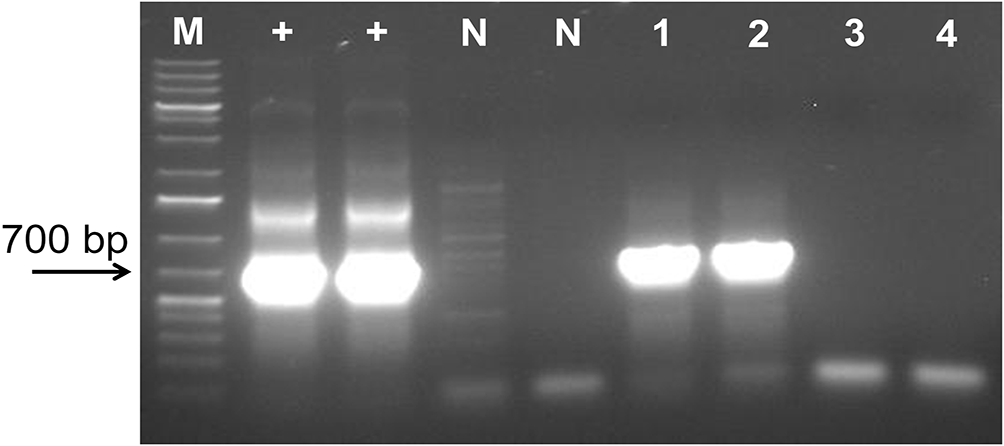

Supplement: Figure S5 — Confirmation of X. fastidiosa carrying pXF20-mqsR. PCR performed with a specific pair of primers to detect the pXF20-mqsR construct in DNA samples extracted from 11399-mqsR. The expected size of the amplicon is 760 bp. M—GeneRuler 1 kb Plus DNA Ladder (Thermo Scientific). +—plasmid miniprep (PureYield Plasmid Miniprep System—Promega) of the pXF20-mqsR construct (positive control). N—PCR reaction carried out with DNA extracted from 11399-WT (negative control). Wells 1 and 2 indicate PCR reaction carried out with DNA extracted from 11399-mqsR. Wells 3 and 4 indicate PCR reaction carried out with MilliQ water to assess possible contamination in the pair of primers. The 760 bp amplicon was only observed in the positive control and 11399-mqsR (wells 1 and 2), confirming the transformation of X. fastidiosa with the pXF20-mqsR construct. [file Image5.TIF]
