# Supplementary material for: The MqsRA Toxin-Antitoxin System from Xylella fastidiosa Plays a Key Role in Bacterial Fitness, Pathogenicity, and Persister Cell Formation
Source: Front Microbiol. 2016 Jun 10;7:904. doi: 10.3389/fmicb.2016.00904 (PMC4901048; doi:10.3389/fmicb.2016.00904)
Supplement: Table S1 — Bacterial strains and plasmids used in this study. [file Table1.DOCX]

**Table S1. Bacterial strains and plasmids used in this study.**

| **Strains and plasmids** | **Genotype/relevant characteristics** | **Source** |
| --- | --- | --- |
| ***X. fastidiosa*** |  |  |
| 11399 | Wild-type | Coletta-Filho *et al.*, 2001 |
| 11399-*mqsR* | 11399 strain bearing the pXF20-*mqsR* plasmid | this study |
| ***E. coli*** |  |  |
| Rosetta (DE3) | F- *ompT hsdS*B(rB- mB-) *gal dcm* pRARE (Cam^R^) | Novagen |
| **Plasmids** |  |  |
| pXF20 | Amp^R^, Km^R^, cloning vector | Lee *et al.*, 2010 |
| pXF20-*mqsR* | Amp^R^, Km^R^, P_native_-*mqsR* | this study |
| pBAD-HisA | Amp^R^, cloning vector | Invitrogen |
| pBAD-His-*mqsR* | Amp^R^, P_araBAD_-*mqsR* | this study |
| pET28a | Km^R^, cloning vector | Novagen |
| pET28a-*mqsA* | Km^R^, P_T7_-*mqsA* | this study |
| pETDuet-1 | Amp^R^, cloning vector | Novagen |
| pETDuet-1-*mqsR*-*mqsA* | Amp^R^, P_T7_-*mqsR*, P_T7_,-*mqsA* | this study |

Cam^R^, chloramphenicol-resistant; Amp^R^, ampicillin-resistant; Km^R^, kanamycin-resistant.
