# Supplementary material for: The MqsRA Toxin-Antitoxin System from Xylella fastidiosa Plays a Key Role in Bacterial Fitness, Pathogenicity, and Persister Cell Formation
Source: Front Microbiol. 2016 Jun 10;7:904. doi: 10.3389/fmicb.2016.00904 (PMC4901048; doi:10.3389/fmicb.2016.00904)
Supplement: Table S2 — Oligonucleotide primers used in this study. [file Table2.DOCX]

**Table S2. Oligonucleotide primers used in this study.**

| **Primers used for construction in cloning vectors** | | | | |
| --- | --- | --- | --- | --- |
| **Name** | | **Sequence (5’-3’)** | | **Restriction Enzyme** |
| *mqsR pXF20* | | F: TTGTCT**AAGCTT**CCAGGGTGACGGTTTTG | | HindIII |
|  |  | R: TGTCT**GGATCC**GACAGTTAACTTCAGGTAC | | BamHI |
| *mqsR pBAD-HisA* | | F: **CTCGAG**ATGGAGAAAGGCACACCC | | XhoI |
|  |  | R: **GAATTC**TCATAACTCCTTGAAGGA | | EcoRI |
| *mqsA* pET28a | | F: TTGTAT**CATATG**AGATGTCCATGCTG | | NdeI |
|  |  | R: TATAA**GGATCC**AGTACCTTTTAACGGTTAGA | | BamHI |
| *mqsR* pETDuet-1 | | F: TGGTGC**GGATCC**GATGGAGAAAGGCACAC | | BamHI |
|  |  | R: CTCATGG**AAGCTT**TCATAACTCCTTGAAGG | | HindIII |
| *mqsA* pETDuet-1 | | F: TGACC**AGATCT**GATGAGATGTCCATGCTGC | | BglII |
|  |  | R: CGG**GGTACC**TTTGAAACTCTTCACTTCG | | KpnI |
| **Primers used in conventional PCR** | | | | |
| **Name** | | **Sequence (5’-3’)** | | |
| *mqsR oriV pXF20* | | F: GAGAAAGGCACACCCCACTA | | |
|  |  | R: CGCAGCATCCTCATGTACC | | |
| RST 31 – Forward | | GCGTTAATTTTCGAAGTGATTCGA | | |
| RST 33 – Reverse | | CACCATTCGTATCCCGGTG | | |
| **Primers used in qPCR** | | | | |
| **LBI gene ID^1^** | **Gene** | | **Sequence (5’-3’)** | **Amplicon (bp)** |
| XFrrnaA16S-1 | *XFrRNA* | | F: GCGGAATTCCTGGTGTAGCA | 100 |
|  |  |  | R: GCTTTCGTGCCTCAGTGTCA |  |
| XF0656 | *gltT* | | F: GCTTCTGCTGGCACATGGTT | 100 |
|  |  |  | R: CATCGCAGCAAAGCTGGAA |  |
| XF2490 | *mqsR* | | F: CTGGCAAGGTCAGGGCTACA | 100 |
|  |  |  | R: GACGTGAGCGCCAAGACAA |  |
| XF2491 | *mqsA* | | F: CCAAGATACGCCGGAAGCT | 65 |
|  |  |  | R: AACACCACCACCGAACAGTTC |  |
| XF1792 | *pilA* | | F: TGGACGGCGACACGTACAT | 118 |
|  |  |  | R: GGGTCGCGAATGACCTCATA |  |
| XF0372 | *pilP* | | F: GTATTCTGCGCAGGGAATGC | 106 |
|  |  |  | R: TTCCAGCGGCTCCTTACGT |  |
| XF2546 | *pilS* | | F: TGCCGAATCGTTGACTTTGTT | 100 |
|  |  |  | R: GAGCTGCGATGCATGTTTGA |  |
| XF0083 | *fimA* | | F: AGCTCGTACACCGTTCACCAT | 80 |
|  |  |  | R: CTGGGCCTGGCTCAAAATAG |  |
| XF2370 | *gumB* | | F: TTACCGTGACTGGTGCAGTG | 112 |
|  |  |  | R: AGACTCGCCAGCGTGTTTAT |  |
| XF0470 | *eal* | | F: AATTGGCACTGGCCACTATG | 113 |
|  |  |  | R: CACATCATGCGAAGGATCAC |  |

^1^http://aeg.lbi.ic.unicamp.br/xf/; restriction enzyme sites are indicated by bold type.
